# Real-time visualization of mRNA synthesis during memory formation in live mice

**DOI:** 10.1073/pnas.2117076119

**Published:** 2022-07-01

**Authors:** Byung Hun Lee, Jae Youn Shim, Hyungseok C. Moon, Dong Wook Kim, Jiwon Kim, Jang Soo Yook, Jinhyun Kim, Hye Yoon Park

**Affiliations:** ^a^Department of Physics and Astronomy, Seoul National University, Seoul 08826, Korea;; ^b^Center for Functional Connectomics, Brain Science Institute, Korea Institute of Science and Technology, Seoul 02792, Korea;; ^c^The Institute of Applied Physics, Seoul National University, Seoul 08826, Korea;; ^d^Department of Electrical and Computer Engineering, University of Minnesota, Minneapolis, MN 55455

**Keywords:** *Arc* mRNA, in vivo imaging, virtual reality, memory trace, engram

## Abstract

Memories are thought to be encoded in populations of neurons called memory trace or engram cells. However, little is known about the dynamics of these cells because of the difficulty in real-time monitoring of them over long periods of time in vivo. To overcome this limitation, we present a genetically encoded RNA indicator (GERI) mouse for intravital chronic imaging of endogenous *Arc* messenger RNA (mRNA)—a popular marker for memory trace cells. We used our GERI to identify *Arc*-positive neurons in real time without the delay associated with reporter protein expression in conventional approaches. We found that the *Arc*-positive neuronal populations rapidly turned over within 2 d in the hippocampal CA1 region, whereas ∼4% of neurons in the retrosplenial cortex consistently expressed *Arc* following contextual fear conditioning and repeated memory retrievals. Dual imaging of GERI and a calcium indicator in CA1 of mice navigating a virtual reality environment revealed that only the population of neurons expressing *Arc* during both encoding and retrieval exhibited relatively high calcium activity in a context-specific manner. This in vivo RNA-imaging approach opens the possibility of unraveling the dynamics of the neuronal population underlying various learning and memory processes.

## Significance

*Arc* is one of the genes that are rapidly transcribed by neuronal activity and thus used as a marker for memory trace or engram cells. However, the dynamics of engram cell populations is not well-known because of the difficulty in monitoring the rapid and transient gene expression in live animals. Using a mouse model in which endogenous *Arc* messenger RNA (mRNA) is fluorescently labeled, we demonstrate that *Arc*-expressing neuronal populations have distinct dynamics in different brain regions and that only a small subpopulation that consistently expresses *Arc* during both memory encoding and retrieval exhibits context-specific calcium activity. This live-animal RNA-imaging technique will offer a powerful tool for connecting gene expression to neuronal activity patterns and to behavior.

Activity-dependent gene expression is critical for long-term memory formation (1). Because immediate-early genes (IEGs) such as *Arc*, *c-Fos*, and *Egr-1* are rapidly and transiently transcribed within a few minutes following stimulation (2), their expression is widely used to identify neurons activated by diverse learning and memory tasks (3). Recent optogenetic and chemogenetic studies have demonstrated that IEG-expressing neurons are involved in formation of so-called memory traces or engrams (4, 5). Thus, IEG-positive neurons are often referred to as engram cells, which are defined by their activation for memory encoding and retrieval (6). However, the overlap between neuronal populations expressing IEGs during encoding and retrieval is relatively low, raising important questions as to whether technical limitations hinder precise identification of engram cells or whether engrams have an inherently dynamic nature (7).

Current methods for imaging IEG expression include RNA fluorescence in situ hybridization (FISH) (2) or immunostaining, and IEG promoter-driven expression of exogenous reporter proteins (4, 8–18). These methods have been used to take snapshots of the neuronal populations activated during memory encoding and retrieval. To date, however, it has not been possible to continuously monitor IEG transcription in real time in live animals because reporter proteins are typically expressed with a delay longer than an hour after transcription (8, 9, 11, 13, 19). For this reason, it is still largely unknown what type of neuronal activity triggers IEG transcription at the single-cell level. Moreover, even short-lived versions of fluorescent proteins decay over several hours to a few days (8, 9, 15, 20). Therefore, it has been difficult to identify distinct IEG-positive neuronal populations that are activated by different behaviors or events at a time interval of less than a day. Various methods using tTA-TetO or tamoxifen-inducible Cre recombinase systems have been used to restrict IEG activity tagging to a particular time window (4, 10, 12, 14, 16, 18), but the duration of the tagging window is typically hours to days. Hence, there have been concerns that these methods may overestimate the size of the neuronal population activated by a single experience (7, 21).

Herein, we report a genetically encoded RNA indicator (GERI) imaging technique that enables the real-time monitoring of endogenous IEG transcription in the living brain. We generated a GERI mouse in which every single endogenous *Arc* messenger RNA (mRNA) was labeled with up to 48 green fluorescent proteins (GFPs) and visualized individual *Arc* transcription sites (TSs) in vivo using two-photon excitation microscopy. By performing in vivo time-lapse imaging and simulations, we demonstrate that GERI reports the rapid and transient transcription of *Arc* mRNA with a high accuracy in real time, which has not been achieved by existing fluorescent protein reporters. Using this unique tool, we found that the *Arc*-positive neuronal populations have distinct dynamic properties in different brain regions. We also demonstrate that GERI can be used in conjunction with a genetically encoded calcium indicator (GECI) to investigate the neuronal activity of *Arc*-positive neurons in awake mice navigating a virtual reality (VR) environment. This work establishes GERI as a versatile tool for probing endogenous transcriptional activity during learning and memory processes in vivo.

## Results

### Characterization of GERI Mice for In Vivo Imaging of *Arc* mRNA.

**Fig. 1.**
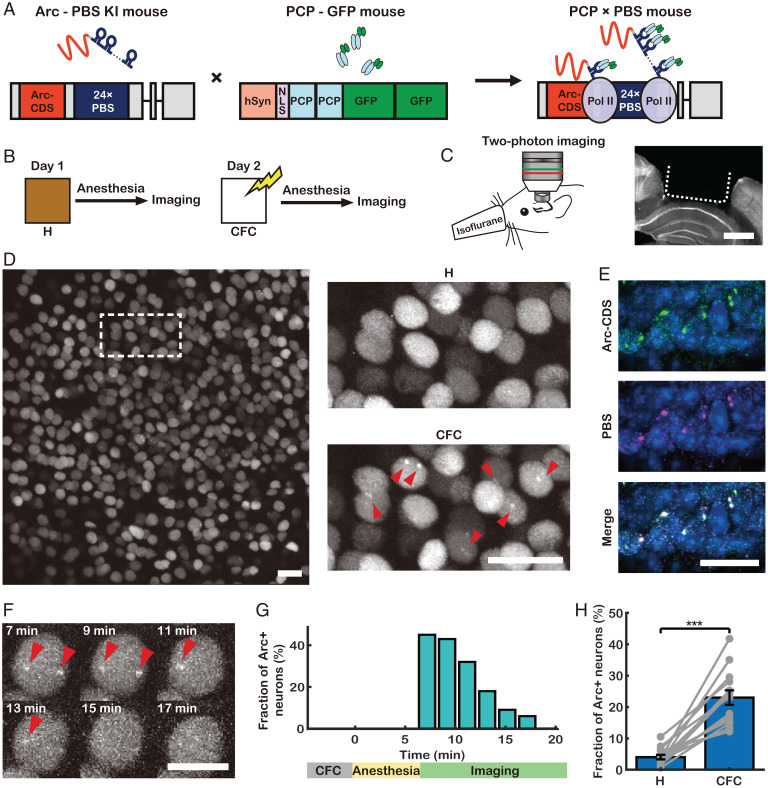
Development of GERI mice for imaging *Arc* mRNA. (*A*) Schematic for labeling *Arc* mRNA in vivo. NLS, nuclear localization sequence; Pol II, RNA polymerase II. Gray boxes, UTR; red boxes, *Arc* coding sequence [*Arc*-CDS]; blue boxes, 24× PBS cassette; black lines, introns. The *Arc*-PBS knockin (KI) mouse was crossed with the PCP-GFP mouse to generate PCP×PBS hybrids. (*B*) On day 1, mice were removed from their home cage (H) and immediately anesthetized for in vivo imaging. On day 2, the mice were subjected to CFC followed by in vivo imaging. (*C*, *Left*) Experimental setup for in vivo two-photon imaging through a hippocampal window. (*C*, *Right*) Coronal view of the brain of a PCP×PBS mouse after hippocampal window surgery. (*D*) Representative in vivo image of CA1 neurons in a PCP×PBS mouse after autofluorescence subtraction (for detailed image-processing procedures, see *SI Appendix*, Fig. S2). The same region (dotted box) is enlarged for comparison of images taken on day 1 (H) and day 2 (CFC). *Arc* TSs are marked with red arrowheads. (*E*) *Arc* mRNA detected by two-color smFISH targeting *Arc*-CDS (green) and PBS (magenta). (*F*) Time-lapse images of an *Arc*+ neuron after CFC. (*G*) Fraction of *Arc*+ neurons over time after CFC. (*H*) Fraction of *Arc*+ neurons in CA1 after H and CFC conditions (*n* = 12 mice; ***P < 10^−4^ by pairwise *t* test). (Scale bars, 1 mm [*C*], 50 μm [*D* and *E*], and 10 μm [*F*].) Error bars represent the SEM.

To label endogenous *Arc* mRNA with GFP, we exploited the highly specific binding between the PP7 bacteriophage capsid protein (PCP) and the PP7 binding site (PBS) RNA stem loop (22). We generated a transgenic mouse that expresses a tandem PCP fused with tandem GFP (PCP-GFP) in neurons. This PCP-GFP mouse was crossed with an *Arc*-PBS mouse, in which 24 PBS repeats were knocked into the 3′ untranslated region (UTR) of the *Arc* gene (23). In the resulting PBS homozygous (*Arc*^P/P^) PCP×PBS hybrid mouse (Fig. 1*A*), every endogenous *Arc* mRNA was labeled with up to 48 GFPs. To confirm that PP7-GFP labeling did not disrupt *Arc* gene expression, we performed Western blotting and immunofluorescence analyses to evaluate the Arc protein expression levels upon fear conditioning in the brains of wild-type (WT), *Arc*-PBS, and PCP×PBS mice, and the results indicated that the Arc protein levels were similar in all mice (*SI Appendix*, Fig. S1 *A*–*D*). Moreover, immunofluorescence analysis in dissociated hippocampal neurons cultured from PCP×PBS mice revealed that neurons with *Arc* TSs expressed significantly higher Arc protein levels than neurons without *Arc* TSs did (*SI Appendix*, Fig. S1 *E*–*G*). This result confirms that *Arc* transcription detected by PCP-GFP was followed by Arc protein expression.

To demonstrate in vivo imaging of *Arc* mRNA, we first investigated *Arc* induction upon contextual fear conditioning (CFC) (Fig. 1*B*). We used two-photon excitation microscopy through a hippocampal window (24) to visualize *Arc* mRNA in the dorsal CA1 region of the PCP×PBS mice (Fig. 1*C*). To minimize motion artifacts and any effects of subsequent handling, we performed in vivo imaging of mice under anesthesia immediately after CFC; previous reports have demonstrated that anesthesia with isoflurane does not cause retrograde amnesia (25, 26). We developed image-processing algorithms for motion correction, autofluorescence subtraction, region of interest (ROI) registration, and automatic segmentation of neurons by their nuclear GFP background originating from the nuclear localization sequence in PCP-GFP (*SI Appendix*, Fig. S2). Using the software, we were able to identify the same neurons across multiple days and weeks. In a subset of neurons, one or two bright spots were clearly visible in the nucleus (red arrowheads, Fig. 1*D*). To confirm that these were *Arc* TSs, we performed two-color single-molecule FISH (smFISH) using probes targeting the coding sequence (CDS) and the PBS sequence of *Arc* mRNA in brain tissues collected after CFC (Fig. 1*E*). *Arc* mRNA particles detected in smFISH images were classified by their size and intensity into two major groups: single mRNAs and TSs. Approximately 93% of the TSs detected by the PBS probe were colocalized with those detected by the CDS probe, and vice versa (*SI Appendix*, Fig. S3). The average copy number of nascent *Arc* mRNAs per TS was estimated to be 15 ± 4 by dividing the intensity of each TS by the average intensity of single mRNAs detected by the PBS probe. Thus, an average of ∼720 GFPs were recruited to each *Arc* locus, providing sufficient signal for three-dimensional (3D) visualization of individual TSs located 100 to 400 μm deep inside the living brain (Movie S1).

To investigate the temporal dynamics of *Arc* transcription, we performed time-lapse imaging and found that the fraction of neurons with *Arc* TSs (*Arc*+ neurons) reached its maximum within 7 min after CFC and then monotonically decreased to basal levels by 20 min after CFC (Fig. 1 *F* and *G*). Under the anesthetized condition, only about 7% of the *Arc*+ neurons showed *Arc* transcription again within 2 h (*SI Appendix*, Fig. S4). Based on these data, the time window selected for in vivo imaging was 4 to 7 min after each behavioral test, substantially faster than the 1 to 3 h after stimulation for existing fluorescent protein reporters (8, 9, 13, 19). To evaluate the performance of GERI compared with a short-half-life GFP (shGFP) reporter, we performed Monte Carlo simulations and assessed the accuracy of classifying IEG-positive and IEG-negative cells. The simulation results showed that the classification accuracy of GERI was superior to that of the shGFP reporter as the basal IEG activation rate was increased (*SI Appendix*, Fig. S5).

Having established GERI imaging and analysis techniques, we measured the size of the *Arc*+ neuronal populations in CA1 of the PCP×PBS mice. We observed *Arc* TSs in 4.1 ± 0.8% of the CA1 neurons in mice taken from their home cages. The percentage of *Arc*+ neurons significantly increased to 23 ± 3% in the same ROIs after CFC on the next day (Fig. 1 *D* and *H*). Three-color smFISH showed that *Arc* and two other IEGs, *c-Fos* and *Egr-1*, were regulated similarly and coexpressed in 19 ± 3% of the neurons in the dorsal CA1 region after CFC (*SI Appendix*, Fig. S6). In addition, we found that *Arc* TSs could be readily detected by GERI without any staining in fixed sections of various brain regions (*SI Appendix*, Fig. S7), which provides another application of GERI for RNA in situ detection.

### Dynamics of the *Arc*+ Neuronal Population in Recent Memory Retrieval.

**Fig. 2.**
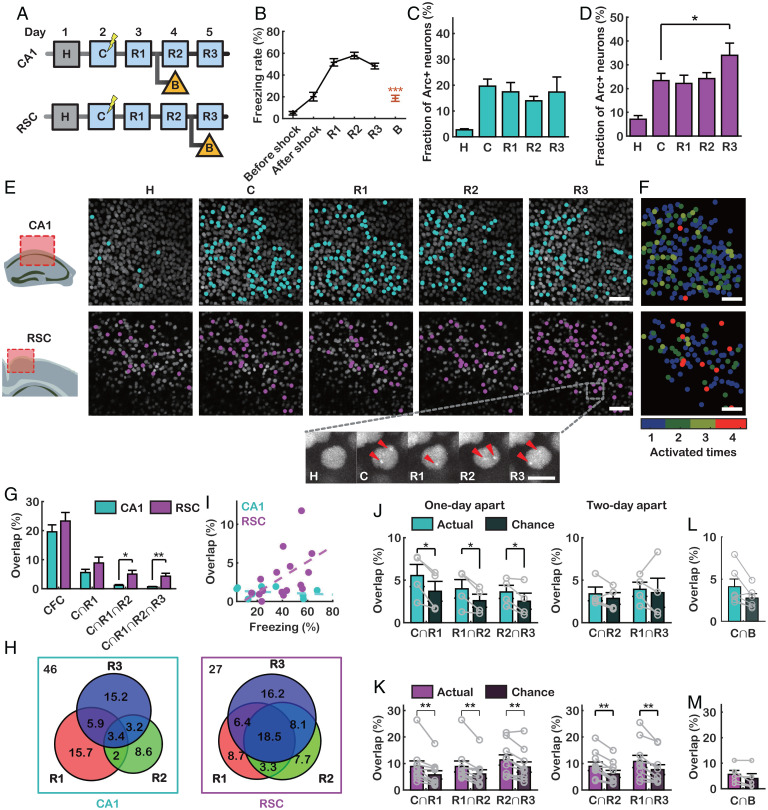
Dynamics of *Arc*+ neuronal populations following CFC and repeated recent memory retrieval. (*A*) Experimental scheme for CFC (C) and repeated retrieval tests (R1 to R3) over 3 consecutive days. Groups of mice were exposed to ctx B on day 4 (CA1) or day 5 (RSC). (*B*) Freezing rates of PCP×PBS mice during CFC and three retrieval tests in ctx A (*n* = 14 mice) and ctx B (*n* = 12 mice) (Tukey’s multiple-comparison test after one-way ANOVA, ***P < 10^−6^). (*C* and *D*) Fraction of *Arc*+ neurons in CA1 (*C*) (*n* = 4 mice) and the RSC (*D*) (*n* = 10 mice) after each behavioral session (*P < 0.05 by pairwise *t* test). (*E*) Representative in vivo images of CA1 (*Top*) and the RSC (*Bottom*), showing the same fields of view after H, C, and R1 to R3. Cyan and magenta dots denote *Arc*+ neurons in CA1 and the RSC, respectively. (*E*, *Bottom*) Example images of a neuron, with red arrowheads indicating *Arc* TSs. (*F*) *Arc*+ neurons in CA1 (*Top*) and the RSC (*Bottom*), colored by the number of times the neurons expressed *Arc*. (*G*) Overlap percentage of consecutively reactivated *Arc*+ neurons in CA1 and the RSC (*P < 0.05, **P < 0.01 by rank-sum test). (*H*) Venn diagrams of neurons that expressed *Arc* during CFC and reexpressed *Arc* during each retrieval test (*Left*: CA1; *Right*: RSC). Numbers indicate percentages of neurons. (*I*) The freezing rate was correlated with the overlap rate of *Arc*+ neurons in the RSC (magenta) but not in CA1 (cyan) (CA1: *R* = −0.24, *P* = 0.75, *n* = 10 mice; RSC: *R* = 0.50, *P* = 0.02, *n* = 16 mice; by Pearson’s correlation; *z* = 1.69, *P* = 0.045 by Fisher’s *z* test for two independent correlations). (*J* and *K*) Overlap percentage of *Arc*+ neurons at 1- (*Left*) or 2-d intervals (*Right*) (*P < 0.05, **P < 0.01 by pairwise *t* test) in CA1 (*J*) and the RSC (*K*). (*L* and *M*) Overlap of *Arc*+ populations between the CFC and ctx B conditions compared with chance in CA1 (*L*) (*P* = 0.056; *n* = 6 mice) and the RSC (*M*) (*P* = 0.12; *n* = 6 mice). (Scale bars, 50 μm [*E* and *F*] and 10 μm [*E*, *Inset*].) Error bars represent the SEM.

Using our in vivo GERI imaging technique, we next investigated how the populations of *Arc*+ neurons change upon memory encoding and retrieval. We performed fear conditioning in context A (ctx A) and repeatedly exposed the mice to ctx A to retrieve fear memory on 3 consecutive days (Fig. 2*A*). The animals showed freezing behavior during all retrieval tests (R1 to R3) in ctx A but not in a new context (ctx B) (Fig. 2*B*), indicating that conditioned context-specific fear memory remained intact. To compare the dynamics of the *Arc*+ neuronal population in different brain regions, we performed cranial window imaging of the dorsal hippocampal CA1 region and the retrosplenial cortex (RSC), which have essential roles in spatial cognition and memory with strong reciprocal connectivity (27–29). A similar fraction (14 to 20%) of neurons in CA1 transcribed *Arc* after CFC and R1 to R3 (Fig. 2*C*). The fraction of *Arc*+ neurons in the RSC was higher than that in CA1 and increased from 23 ± 3% (CFC) to 34 ± 5% (R3) (Fig. 2*D*).

Taking advantage of our live-animal imaging, we performed longitudinal analysis of *Arc* transcription in individual neurons. We found that a distinct population of neurons expressed *Arc* mRNA every time contextual memory was retrieved (Fig. 2*E*). We then identified the overlapping population of *Arc*+ neurons across the CFC and R1 to R3 (*SI Appendix*, Fig. S8*A*). A small population of neurons persistently showed *Arc* transcription throughout both the conditioning and retrieval sessions (Fig. 2 *E*, *Bottom*). This persistently overlapping population (red dots, Fig. 2*F*) represented 4.3 ± 1.0% of the neurons in the RSC, which was significantly higher than that in CA1 (0.6 ± 0.1%) (Fig. 2*G*). The population of neurons that expressed *Arc* after CFC and each retrieval session also overlapped more in the RSC than in CA1 (Fig. 2*H*). Moreover, we found a positive correlation between the freezing rate and the fraction of the persistently overlapping *Arc*+ population in the RSC, whereas there was no such correlation in CA1 (Fig. 2*I*). Since the freezing rate reflects the strength of fear memory, the persistently overlapping *Arc*+ neurons in the RSC could be a stable component of contextual fear memory (10). However, there were more dynamic changes occurring in the *Arc*+ neuronal population in CA1 during memory encoding and retrieval.

To assess the dynamics of *Arc* reexpression, we compared the overlap of *Arc*+ neurons upon reexposure to ctx A at 1- or 2-d intervals with that expected by chance. In CA1, the overlap was greater than expected by chance in the 1-d-apart comparison but equivalent to random in the 2-d-apart comparison (Fig. 2*J*). When the first retrieval (R1) was performed at 2 d after CFC, the overlap was also similar to random chance (*SI Appendix*, Fig. S8 *B* and *C*), which suggests that this drop-off in overlapping neurons is dependent on time rather than the number of retrievals. However, in the RSC, the overlap in both 1- and 2-d-apart comparisons was significantly greater than that expected by chance (Fig. 2*K*). We confirmed that the degree of overlap between the *Arc*+ neurons activated during CFC and those activated upon exposure to ctx B was almost random in both CA1 and the RSC (Fig. 2 *L* and *M*). These data indicate that there was a near-complete turnover of *Arc*+ neurons within 2 d in CA1, consistent with previous reports (15, 30–32). In contrast, the *Arc*+ neurons in the RSC exhibited either slower dynamics or greater stability.

### Stability of the Overlapping *Arc*+ Population in Remote Memory Retrieval.

**Fig. 3.**
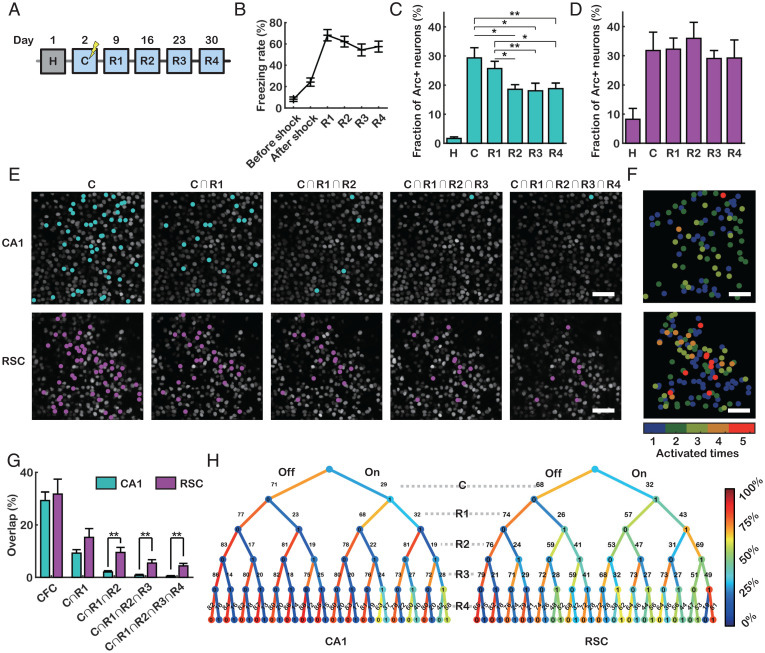
Long-term dynamics of *Arc*+ neuronal ensembles in CA1 and the RSC. (*A*) Experimental scheme for CFC (C) and remote memory retrieval tests (R1 to R4). (*B*) Freezing rates of PCP×PBS mice during CFC and four retrieval tests (*n* = 11 mice). (*C* and *D*) Fraction of *Arc*+ neurons in CA1 (*C*) (*n* = 6) and the RSC (*D*) (*n* = 5) after each session (*P < 0.05, **P < 0.01 by pairwise *t* test). (*E*) Representative images of CA1 (*Top*) and the RSC (*Bottom*) showing overlapping populations of *Arc*+ neurons (cyan, CA1; magenta, RSC). (*F*) *Arc*+ neurons in CA1 (*Top*) and the RSC (*Bottom*) labeled to reflect the number of *Arc* expression events. (*G*) The percentage of neurons that consecutively reexpressed *Arc* in CA1 (cyan) and the RSC (magenta) (**P < 0.01 by Student’s *t* test). (*H*) Tree graphs colored by the reexpression probability of neurons in CA1 (*Left*) and the RSC (*Right*). The first layer represents the fraction of neurons that did (rightward) or did not express (leftward) *Arc* following CFC. The other layers are further split according to the presence or absence of *Arc* TSs following memory retrieval. (Scale bars, 50 μm [*E* and *F*].) Error bars represent the SEM.

To examine the stability of *Arc*+ neuronal ensembles over longer timescales, we performed repeated retrieval experiments on days 9, 16, 23, and 30 (Fig. 3*A*). All fear-conditioned mice showed freezing behaviors during the remote memory retrieval tests (Fig. 3*B*). The fraction of *Arc*+ neurons in CA1 decreased significantly after 2 wk (Fig. 3*C*), whereas the fraction in the RSC was similar (29 to 36%) for all sessions (Fig. 3*D*). The overlapping population of *Arc*+ neurons sharply decreased in CA1 but remained stable in the RSC for at least 1 mo (Fig. 3*E*). Persistent *Arc* reexpression up to the fourth retrieval session was observed in 4.4 ± 1.0% of the RSC neurons but in almost none (0.4 ± 0.3%) of the CA1 neurons (Fig. 3*F*). The consecutive overlap of *Arc*+ neurons was significantly higher in the RSC than in CA1 from R2 (Fig. 3*G*). The overlap between the *Arc*+ populations elicited by memory retrieval 1 to 4 wk apart was also higher than that expected by chance in the RSC but not in CA1 (*SI Appendix*, Fig. S9). To visualize how *Arc*+ neurons turn over during remote memory retrieval, we used tree graphs to trace *Arc* reexpression probabilities (Fig. 3*H*). RSC neurons that consistently expressed *Arc* were more likely to be reactivated, in accordance with previous reports on neocortical IEG expression (9, 18, 19, 27, 33). However, in CA1, an almost random population of neurons expressed *Arc* whenever the mouse was reexposed to the same context.

### Calcium Activity of *Arc*+ Neurons during VR Navigation.

**Fig. 4.**
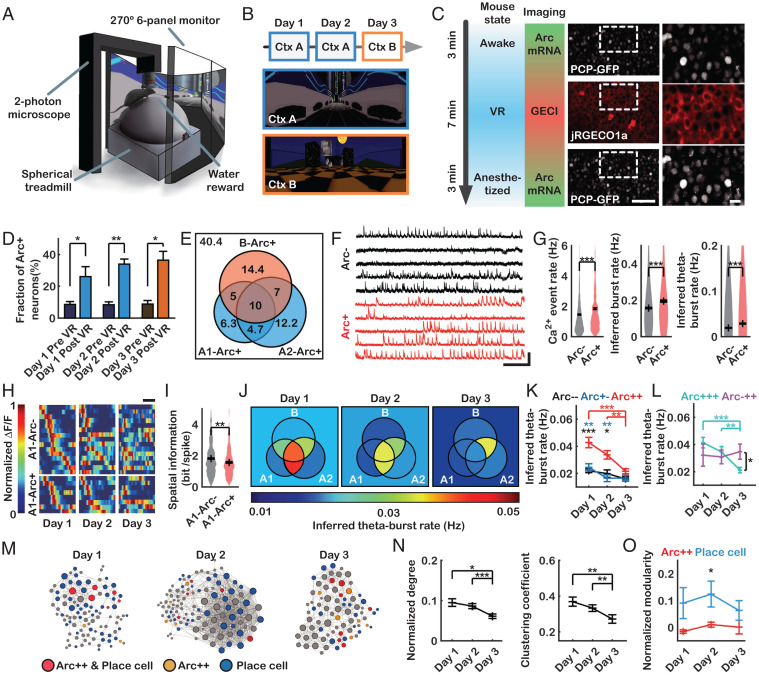
*Arc* transcription and calcium imaging in CA1 of mice exploring VR. (*A*) Experimental setup. (*B*) Experimental schedule (*Top*) and virtual contexts A (*Middle*) and B (*Bottom*). (*C*) *Arc* transcription imaging was first conducted while the mice were awake, followed by calcium imaging while exploring VR. After 7 min of exploration, *Arc* transcription imaging was performed under anesthesia. Representative images and magnified insets are shown (*Right*). (*D*) Fractions of neurons with *Arc* TSs before and after VR on each day (*P < 0.05, **P < 0.01 by pairwise *t* test; *n* = 6 mice). (*E*) A Venn diagram of *Arc*+ neurons on days 1, 2, and 3, showing the percentage of each subpopulation. (*F*) Representative calcium traces of *Arc*− and *Arc*+ neurons. (*G*, *Left*) Ca^2+^ event rates of *Arc*− and *Arc*+ neurons (***P < 10^−8^ by rank-sum test). (*G*, *Middle*) Inferred burst rates of *Arc*− and *Arc*+ neurons (***P < 10^−10^ by rank-sum test). (*G*, *Right*) Inferred theta-burst rates of *Arc*− and *Arc*+ neurons (***P < 10^−19^ by rank-sum test) (*Arc*−: *n* = 1,085 neurons; *Arc*+: *n* = 651 neurons). (*H*) Example of place fields of A1-*Arc*− (*Top*) or A1-*Arc*+ (*Bottom*) place cells on days 1, 2, and 3. Neurons are ordered by the locations of the place field that yielded the maximum activity on day 1. (*I*) Spatial information of A1-*Arc*− and A1-*Arc*+ neurons on day 1 (**P < 0.01 by rank-sum test; A1-*Arc*−: *n* = 430 neurons; A1-*Arc*+: *n* = 209 neurons). (*J*) Venn diagrams of average inferred theta-burst rates of each subpopulation on days 1, 2, and 3. (*K*) Inferred theta-burst rates of *Arc*−−, *Arc*+−, and *Arc*++ neurons on each day (*P < 0.05, **P < 0.01, ***P < 10^−4^ by Student’s *t* test; *Arc*−−: *n* = 261 to 302 neurons; *Arc*+−: *n* = 69 to 92 neurons; *Arc*++: *n* = 103 to 116 neurons). (*L*) Inferred theta-burst rate of *Arc*−++ and *Arc*+++ neurons on each day (*P < 0.05, **P < 0.01, ***P < 10^−4^ by Student’s *t* test; *Arc*−++: *n* = 36 to 47 neurons; *Arc*+++: *n* = 65 to 81 neurons). (*M*) Example network graphs from a mouse on days 1, 2, and 3. Nodes are classified by color, and node size is proportional to the degree of the neurons. (*N*) Normalized degree (*Left*) and clustering coefficient (*Right*) of *Arc*++ neurons on each day (*P < 0.05, **P < 0.01, ***P < 10^−4^ by rank-sum test; *n* = 103 to 116 neurons). (*O*) Normalized modularity when dividing neurons into place cells and nonplace cells (blue) and dividing neurons into *Arc*++ and non-*Arc*++ (red) (*P < 0.05 by Student’s *t* test; *n* = 6 mice). (Scale bars, 50 μm [*C*, *Middle*], 10 μm [*C*, *Right*], 1 min [*F*, horizontal], 150% Δ*F*/*F* [*F*, vertical], and 1 m [*H*].) Error bars represent the SEM.

Given our data on the high turnover rate of *Arc*+ neurons in CA1, we next sought to investigate the neuronal activity of these cells during memory encoding and retrieval. To monitor the calcium activity of *Arc*+ neurons, we performed dual-color imaging of CA1 in head-fixed awake mice running on a trackball to navigate a linear track in VR (Fig. 4*A*). We injected an adeno-associated virus (AAV) expressing jRGECO1a (34) into the dorsal CA1 region of the PCP×PBS mice (*SI Appendix*, Fig. S10). Six mice were exposed to a novel context (ctx A) on day 1 and again on day 2, followed by a distinct context (ctx B) on day 3 (Fig. 4*B*). Each day, we imaged *Arc* mRNA in the awake resting state, calcium activity during VR navigation, and *Arc* mRNA under anesthesia (Fig. 4*C*, *SI Appendix*, Fig. S11, and Movie S2). The fraction of neurons with *Arc* TSs significantly increased after VR navigation each day (Fig. 4*D*). The calcium activity of the *Arc*+ neurons was similar regardless of the prior transcription of *Arc* before VR navigation (*SI Appendix*, Fig. S12). Cells that transcribed *Arc* after VR navigation on days 1, 2, and 3 were referred to as A1-*Arc*+, A2-*Arc*+, and B-*Arc*+ neurons, respectively; the overlap between the three *Arc*+ populations was 15 to 17% (Fig. 4*E*)—significantly greater than expected by chance (*SI Appendix*, Fig. S13). We next compared the calcium activity of the *Arc*+ and *Arc*− neurons pooled from all three sessions. The calcium traces were deconvolved to infer spike trains (35), from which we calculated the “inferred burst” and “inferred theta-burst” (6 to 10 Hz) rates (*SI Appendix*, Fig. S12). By applying the same analysis to cultured neurons under electrical stimulation, we confirmed that burst activity at a frequency of 6 to 10 Hz could be detected by using this deconvolution algorithm (*SI Appendix*, Fig. S14). Although almost all neurons (99.9%) showed varying degrees of calcium activity (Fig. 4*F*), we found that *Arc*+ neurons were significantly more active than *Arc*− neurons on average, in terms of calcium event, inferred burst, and inferred theta-burst rates (Fig. 4*G* and *SI Appendix*, Fig. S12).

Next, we identified place cells on each day using a similar method previously reported (24) and calculated the spatial correlation between the corresponding place fields (Fig. 4*H* and *SI Appendix*, Fig. S15 *A*–*C*). The correlation between place fields in the same context (A–A) was significantly higher than that in different contexts (A–B), indicating that the mice perceived the two virtual environments as different contexts (*SI Appendix*, Fig. S15*D*). Among the place cells, A1-*Arc*+ neurons showed weaker spatial correlations between days 1 and 2 than A1-*Arc*− neurons did (*SI Appendix*, Fig. S15*E*). A1-*Arc*+ neurons also had lower spatial information than A1-*Arc*− neurons on day 1 (Fig. 4*I*), consistent with a previous report on *c-Fos*–positive neurons (36). These results support the notion that IEG-positive CA1 neurons do not necessarily represent spatial information about the context but rather serve as an index to episodic memory (37).

To further dissect the activity of *Arc*+ neurons, we grouped the CA1 neurons into eight subpopulations, as shown in Fig. 4*E*, and compared their calcium activity on each day (Fig. 4*J* and *SI Appendix*, Fig. S16). Neurons that transcribed *Arc* on both days 1 and 2 (*Arc*++), which includes both *Arc*+++ and *Arc*++− neurons, had higher inferred theta-burst activity in ctx A than in ctx B (Fig. 4*K*). Neurons that expressed *Arc* on day 1 but not on day 2 (*Arc*+−) showed a similar inferred theta-burst rate as neurons that did not express *Arc* on either day (*Arc−−*) throughout days 1 to 3 (Fig. 4*K*). Both *Arc*+− and *Arc−−* neurons exhibited significantly lower inferred theta-burst activity than *Arc*++ neurons on days 1 and 2 (Fig. 4*K*). These data suggest that only *Arc*++ neurons have a particular activity pattern associated with ctx A. Interestingly, neurons that expressed *Arc* on all 3 d (*Arc*+++) also showed significantly higher inferred theta-burst rates in ctx A than in ctx B (Fig. 4 *J* and *L*), indicating more association with ctx A than ctx B. On the other hand, neurons that expressed *Arc* only on days 2 and 3 (*Arc*−++) showed a high inferred theta-burst rate on day 3 (Fig. 4 *J* and *L*). When the order of exposure to ctx A and B was reversed on days 2 and 3, only the *Arc*+− subpopulation exhibited higher theta-burst activity in ctx A (on days 1 and 3) than in ctx B (on day 2) (*SI Appendix*, Fig. S17). Taken together, these results suggest that only a subset, not all, of *Arc*+ neurons show a specific activity pattern associated with the exposed context.

We then examined whether the overlapping *Arc*++ neurons were simultaneously reactivated during memory retrieval as in optogenetic experiments (37). The correlation coefficients among the *Arc*++ neurons were similar to those among the non-*Arc*++ neurons (*SI Appendix*, Fig. S16 *B* and *C*). After calculating the correlation coefficient matrices, we generated network graphs of the correlated neuronal activity on each day (Fig. 4*M*). The network properties were quantified using the number of correlated pairs (degree), the fraction of pairs among neighbors (clustering coefficient) (38), and the strength of division of the network into modules (modularity) (39). *Arc*++ neurons showed a higher normalized degree and clustering coefficient in ctx A than ctx B (Fig. 4*N*), indicating that their activity was highly correlated with that of other CA1 neurons during encoding and retrieval of contextual memory. The modularity of *Arc*++ neurons was close to zero (Fig. 4*O*), suggesting that they were integrated with rather than segregated from other neurons in CA1. Meanwhile, the place cells identified each day showed higher modularity than the *Arc*++ neurons (Fig. 4*O*) and formed a dense cluster on day 2 (Fig. 4*M*), consistent with a previous report (40). These results suggest that *Arc*++ neurons do not necessarily have synchronized inputs but rather exhibit correlated activity with other CA1 neurons in a context-specific manner.

## Discussion

Our GERI imaging technique enables the real-time visualization of endogenous IEG transcription at individual gene loci in live animals. Unlike conventional reporter protein expression approaches, GERI can directly report the rapid and transient transcription of IEGs with high accuracy. Using this unique tool, we found that the *Arc*+ neuronal populations in the hippocampus and the cortex have distinct dynamic properties. In CA1, a similar fraction (∼20%) of neurons expressed *Arc* during both recent and remote memory retrieval, but the individual neurons involved rapidly changed within 2 d (Fig. 2*J*). However, in the RSC, a small yet significant fraction (∼4%) of neurons persistently expressed *Arc* during encoding and in every retrieval session for at least 1 mo (Fig. 3*G*). The proportion of this persistent *Arc*+ population in the RSC was correlated with the freezing rate (Fig. 2*I*), indicating a role for these stable *Arc*+ neurons in contextual fear memory. These findings are particularly interesting in the context of memory-indexing theory, in which hippocampal ensembles are proposed to serve as indices whereas cortical ensembles serve as content (41). From this perspective, our results suggest that each hippocampal *Arc*+ population represents a new or updated index for each retrieval event, while the stable RSC *Arc*+ population contains information about context (37). Moreover, the drastic decrease in the overlapping *Arc*+ population in CA1 implies that the role of the hippocampus in systems memory consolidation is limited to short timescales of a few days (6, 42, 43).

Although IEG expression has long been used as a marker for recent activity (2), the exact relationship between neuronal activity and IEG transcription at the single-cell level has not been established. By combining GERI with GECI imaging in awake mice navigating a VR environment, we found that the *Arc*+ population had higher average calcium activity than the *Arc*− population, but individual neurons in both groups showed widely varying degrees of calcium activity (Fig. 4 *F* and *G*). Only the overlapping *Arc*++ subpopulation in CA1 consistently exhibited higher inferred theta-burst activity than other neurons during both memory encoding and retrieval (Fig. 4 *J* and *K*), suggesting that the *Arc*++ population, rather than the whole *Arc*+ population, may be involved in contextual memory.

Because *Arc* is a key regulator of synaptic plasticity (44), the transcription and translation of *Arc* upon the initial exposure to the fear conditioning context might be sufficient to form the memory engram. *Arc* expression during memory retrieval may rather have a role in modifying or updating the representation of memory. From this perspective, an alternative interpretation is possible, in which the neurons that express *Arc* during encoding but not during retrieval (*Arc*+−) could be the neuronal population that maintains the original memory. However, we found that *Arc*+− neurons showed similar levels of low-calcium activity as neurons that never expressed *Arc* (*Arc*−−) throughout days 1 to 3 in our VR experiment (Fig. 4 *J* and *K*). Therefore, our data support the interpretation that the overlapping *Ar*c++ but not the *Arc*+− subpopulation is involved in the encoding and retrieval of contextual memory. Consistent *Arc* expression across reexposures probably has a role in synaptic homeostasis and/or reconsolidation to stabilize the memory trace (3, 44). Further technical developments for the selective manipulation of subpopulations such as *Ar*c++ will enable more precise identification of memory trace or engram cells.

The live-animal RNA-imaging approach demonstrated here provides a powerful platform for future experiments to elucidate the dynamics of memory formation in conjunction with various calcium or voltage indicators, biosensors (45), and optogenetic tools (46). Moreover, different GERI tools could be designed using newly developed background-free RNA-labeling systems such as MS2-PP7 (47, 48) to achieve a higher signal-to-noise ratio for RNA imaging in freely behaving mice. Ultimately, we expect GERI to enable visualization of even single RNA molecules in subcellular compartments such as dendritic spines for studies of RNA localization (23, 49, 50) in the live brain.

## Materials and Methods

### Generation of the PCP-GFP Transgenic Mouse Line.

Animal care practices and all experimental protocols were approved by the Institutional Animal Care and Use Committee at Seoul National University. We generated a transgenic mouse line that expresses a synonymous tandem PP7 capsid protein fused with tandem GFP (stdPCP-stdGFP) (51) under the control of the human synapsin-1 promoter (*hSyn*). The *hSyn-stdPCP-stdGFP* transgene was flanked by two *Rosa26* arms, and the resulting DNA fragments were microinjected into zygotes from C57BL/6N WT mice. The transgene integration site was found between positions 26,509,464 and 26,536,787 on chromosome 13 by whole-genome sequencing.

### Genotyping.

Genotyping of PCP-GFP transgenic mice was performed by PCR analysis using the following primer sets. For the PCP-GFP allele, we used the forward primer hSyn_F (5′-CGACTCAGCGCTGCCTCAGTCT-3′) and reverse primer PCP_R (5′-CGTGTATCTAACCTTAGGTAGACC-3′), yielding a 383-bp product. For the WT allele, we used the forward primer Ch13_F (5′-GTCTAGAGTGCTGCTTGTCTCC-3′) and reverse primer Ch13_R (5′-CTGTGCTTCAAAACCCCATGACC-3′), yielding a 733-bp product. PCR was conducted at 95 °C for 30 s, 60 °C for 30 s, and 72 °C for 60 s for 35 cycles.

PCP-GFP and *Arc*-PBS knockin mice were cross-bred (23) to obtain a double-homozygous PCP×PBS hybrid mouse line. For genotyping of the *Arc*-PBS knockin, we performed PCR analysis using the following primer sets. For the 5′ end, we used two forward primers, Arc PBS gt 5F (5′-TGTCCAGCCAGACATCTACT-3′) and Arc PBS gt 5R (5′-TAGCATCTGCCCTAGGATGT-3′), and one reverse primer, PBS scr R1 (5′-GTTTCTAGAGTCGACCTGCA-3′), yielding a 320-bp product for the WT *Arc* allele and a 228-bp product for the PBS knockin allele. For the 3′ end, we used the forward primer Arc PBS gt 3F (5′-GACCCATACTCATTTGGCTG-3′) and reverse primer Arc PBS gt 3R (5′-GCCGAGGATTCTAGACTTAG-3′), yielding a 332-bp product for the WT *Arc* allele and a 413-bp product for the PBS knockin allele. PCR was conducted at 94 °C for 30 s, 55 °C for 30 s, and 72 °C for 30 s for 35 cycles.

### Cranial Window Surgery and Virus Injections.

For in vivo imaging experiments, we used 8- to 11-wk-old male PCP×PBS hybrid mice that were heterozygous for PCP-GFP and homozygous for the *Arc*-PBS knockin. Mice were anesthetized by intraperitoneal injection of ketamine/xylazine and fixed on a stereotactic frame. Chronic hippocampal windows were implanted as described previously (24). Briefly, a 2.7- to 2.8-mm-diameter craniotomy was made centered at 2.0 mm posterior and 1.8 mm lateral to the bregma by using a 2.7-mm-diameter trephine drill (FST) overlying the dorsal hippocampus. The dura was removed with forceps, and the cortical tissue above the CA1 region was carefully removed by aspiration. A customized stainless-steel cylindrical cannula with a 2.5-mm Ø round glass coverslip (Marienfeld, custom order) attached at the bottom was inserted and cemented to the skull using Meta-Bond (Parkell). Chronic cranial windows over the RSC were implanted at 1.6 mm posterior, 0.5 mm lateral from the bregma. A 2.5-mm Ø round glass coverslip was placed on the craniotomy and sealed with Meta-Bond. Before the Meta-Bond was cured, a customized stainless-steel head ring was placed around either the cannula or the window, and fixed by adding more Meta-Bond.

For VR experiments, we injected 1 μL of 10^13^ viral genomes per milliliter of AAV1-hSyn-NES-jRGECO1a (Addgene, 100854) into 2.0 mm posterior, 1.8 mm lateral, and 1.4 mm ventral to the bregma. Three days after virus injection, hippocampal window surgery was performed. The injection was made using a 33-gauge needle connected to a Hamilton syringe and mounted on a microinjection pump (Harvard Apparatus, Pump 11 Pico Plus Elite).

### CFC Experiments.

After cranial window surgery, mice were housed in the home cage for 7 d and habituated by daily handling and exposure to the anesthesia induction chamber for 5 min. On the eighth day (day 1 of the experiment), the mice were removed from the home cage and imaged under anesthesia (∼1% isoflurane). On day 2, CFC was performed in ctx A, which was a 250 × 250 × 250 mm acrylic box with white and black walls with yellow stripes. The chamber was equipped with a stainless-steel grid floor, a light-emitting diode, and a video camera (Canon, EOS Hi). The mice explored ctx A for 180 s, and three 0.75-mA foot shocks of 2-s duration were delivered at 30-s intervals. From day 3, mice were returned to the conditioning chamber (ctx A) or placed in a different context (ctx B) for 180 s to assess freezing behavior induced by fear memory recall. Ctx B was an equilateral triangular chamber (360 mm per side) scented with 1% acetic acid. Contexts were cleaned with 70% ethanol before each session.

To assess freezing rates, we followed a method described previously (52), and wrote a custom MATLAB script that segments the mouse body and calculates the area of nonoverlapping regions between each pair of consecutive images. If the nonoverlapping area was below a threshold value for at least 0.5 s, the behavior was considered to be “freezing.” The threshold value was adjusted until the freezing rate matched the manually obtained value.

### VR Experiments.

Water restriction and training were performed before VR experiments. Water restriction (1 mL/d) was applied for 5 d using a water dispenser connected to a multichannel syringe pump (New Era, NE-1600). VR experiments were performed using the JetBall-TFT System (Phenosys), which consists of a 270° six-panel monitor, a spherical treadmill, and a water reward device. To restrict the spherical treadmill movement to one dimension, a needle was inserted on one side of the treadmill. After 5 d of water restriction, training was performed on an infinite virtual linear track for ∼0.5 to 1 h/d for 10 to 14 d, and water rewards were delivered at random positions. Mice were subjected to experimentation on the day following their demonstrated capability to travel farther than 50 m in 30 min. On experimental days 1 and 2, mice were exposed to virtual ctx A (Fig. 4*B*) which was 3 m long, 0.4 m wide, and consisted of buildings and moon objects. On day 3, mice were placed into virtual ctx B which was 3 m long, 1 m wide, and made up of objects and wall patterns different from ctx A. After reaching the end of the track, the mice were teleported to the starting point. To induce a perception of movement on a continuous track, tunnels were placed at the front and the end of the virtual contexts. Water reward was delivered at the end of each tunnel.

### In Vivo Two-Photon Imaging.

In vivo imaging was performed using a two-photon excitation laser scanning microscope (Olympus, FVMPE-RS) equipped with two GaAsP photomultiplier tubes (PMTs), a Ti:sapphire laser (Mai-Tai DeepSee, Spectra-Physics), a galvo/resonant scanner, and a 25 × 0.95 numerical aperture water immersion objective with an 8-mm working distance (Olympus, XLSLPLN25XSVMP2). Excitation wavelengths of 900 nm (for *Arc* transcription imaging) and 1,030 nm (for jRGECO1a imaging) were used, and fluorescence was collected via two PMTs after passing through a filter cube (Olympus, FV30-FGR) that consists of a 570-nm low-pass dichroic mirror and two emission filters (495- to 540-nm and 575- to 645-nm band-pass filters). For *Arc* transcription imaging after CFC, mice were anesthetized immediately after each behavior session by 5% isoflurane inhalation using a low-flow vaporizer (Kent Scientific, SomnoSuite) and mounted on the two-photon microscope. Anesthesia was maintained during imaging with ∼1 to 1.5% isoflurane, and body temperature was maintained at 37 °C. We scanned a volume of 250 × 250 × 20 μm at 1,024 × 1,024 × 81 voxels with a scan speed of 2 μs per pixel using a galvo scanner. To find the same ROI again later, we marked ∼2 to 4 spots by laser ablation.

For *Arc* transcription and calcium imaging in VR experiments, mice were mounted on the microscope while awake. *Arc* transcription imaging was performed for ∼3 min before and after the VR exploration. We imaged two ROIs with a volume of 128 × 128 × 20 μm using a resonance scanner. During the VR exploration, calcium activity was observed via jRGECO1a signal. Images of 256 × 256 μm areas were acquired at 30 Hz using a resonance scanner. Calcium imaging was initiated by triggering a signal from JetBall software to simultaneously record VR positions and calcium activity. After the VR exploration, we immediately anesthetized the mice to prevent additional stimulation and imaged *Arc* transcription.

### Image Analysis and Calcium Signal Extraction.

For *Arc* mRNA imaging after CFC, image registration and analysis were performed with custom-written MATLAB codes (*SI Appendix*, Fig. S2). The MATLAB scripts used for image registration and analysis are available at GitHub (https://github.com/Neurobiophysics). Motion artifacts from breathing and cardiac function were corrected by using an autofluorescence image (red channel) as a reference. Images taken on different days were aligned by rotating and translating the images. After the alignment, we subtracted the red-channel image from the green-channel image after normalization by maximum values to remove autofluorescence signals. In our PP7-GFP system, the nuclear localization sequence was added to PCP-GFP to facilitate the identification of neuronal nuclei by the GFP signal. The 3D coordinates of the nuclear centroids were determined by using a circle-finding algorithm (53) in the *XY* and *XZ* plane. By examining the *z* section of each cell image, two experimenters blindly classified the neurons into three groups: neurons with *Arc* TSs (*Arc*+), neurons without *Arc* TSs (*Arc*−), and not-determined (ND) cells. Neurons that showed one or two bright spots in the same *XY* position in at least two consecutive *z* slices were classified as *Arc*+ cells. ND cells include cropped cells and cells that had too high or too low PCP-GFP expression level to detect TSs. Any disagreement between the two experimenters was resolved by a third person. For the analysis of time-lapse imaging of TSs (Fig. 1*G* and *SI Appendix*, Fig. S5*A*), HybTrack software (54) was used to measure the fluorescence intensity of TSs.

For the analysis of immunohistochemistry, the 3D coordinates of the cell centroids were determined by using a circle-finding algorithm (53). Arc protein-positive and -negative neurons were manually classified by the brightness of Arc protein signal. Then, the Arc protein intensity threshold was determined by fitting the intensity histograms of Arc-positive and -negative cells with normal distributions. Then, all brain slices were analyzed based on the same threshold.

Particles in dual-color smFISH images were detected using FISH-quant software (55). FISH-quant provided subpixel positions and intensity by performing 3D Gaussian fitting. A custom-written MATLAB script classified particles into single mRNAs and TSs by setting thresholds for amplitude and width of 3D Gaussian function (*SI Appendix*, Fig. S3*C*). Threshold values were adjusted by examining each slice image manually. TSs detected in both Atto 550 dye (CDS target) and Atto 647 dye (PBS target) channels within 0.3 μm were considered identical (*SI Appendix*, Fig. S3*D*). To quantify the number of nascent mRNAs in a TS, we calculated the intensity of particles by I = Amplitude * σ_x_ * σ_y_ * σ*_z_*, which is an integrated value of a 3D Gaussian function, and divided the TS intensity by the median intensity of single mRNAs.

The image registration and data extraction process for the VR experiment included 1) motion correction of the *Arc* mRNA images using NoRMCorre (56) and StackReg (57) software; 2) stitching two ROIs and subtracting autofluorescence; 3) alignment of images taken before and after VR and on different days; 4) matching the same field of view in *Arc* mRNA and calcium images; 5) obtaining nuclear centroid coordinates using Imaris software (Bitplane); 6) calcium source extraction using CaImAn software (58), which is based on a constrained nonnegative matrix factorization algorithm by seeding the nuclear centroid locations as initial spatial components; and 7) matching the nuclei in *Arc* mRNA images and the spatial components detected by CaImAn by searching the nearest centroid position.

To detect TSs that are near diffraction-limited spots, we need a high signal-to-noise ratio, high spatial resolution, and little distortion from motion artifacts. For this, we acquired 45 images in each *z* plane at 30 Hz and obtained the average image after image registration by *x*-*y* translation using NoRMCorre. The *z* position of the image was then aligned using StackReg software. Two ROIs were stitched using the stitching plugin (59) in ImageJ software. To align stitched voxels taken on different days, we wrote a custom MATLAB script that finds overlapping voxels by calculating cross-correlations (*SI Appendix*, Fig. S11). The calcium activity images were first corrected for motion artifacts by using NoRMCorre software. Cross-correlations were then calculated between the *Arc* mRNA images and averaged calcium images to find the same field of view and corresponding *z* plane. To obtain the 3D coordinates of neuronal nuclei, we used the surface segmentation tool of Imaris (Bitplane) software. The locations of cells within 10 μm of the calcium imaging plane were used as the initial spatial components in CaImAn software. After running CaImAn, the output spatial components were used to identify nuclear coordinates by finding the nearest-neighbor centroid position. The calcium activity traces were calculated using the temporal component values from CaImAn, and relative changes (Δ*F*/*F*) were computed. The calcium trace baseline was selected manually and corrected for photobleaching. A calcium transient event was defined as an event starting from the moment Δ*F*/*F* exceeded 3 SDs of the baseline to the moment that Δ*F*/*F* returned to within 0.5 SD of the baseline. We considered cells with at least one calcium transient to be active cells. False-positive calcium transients due to motion artifacts in the *z* axis were occasionally observed during murine grooming behavior. To remove these artifacts, we used the calcium activity data recorded only during walking (speed > 0.5 cm/s and run length > 0.5 cm). To resolve finer spiking activity, we deconvoluted Δ*F*/*F* traces and inferred the spike traces (35). Ca^2+^ event rates were calculated by dividing the sum of the inferred spikes by time. A burst was defined as spikes that were larger than a threshold value of 2.5 in a 33-ms time bin or continuous over multiple time bins. We defined theta-burst events as bursts that occurred at interburst intervals of 100 to 167 ms (6 to 10 Hz) following a similar method in ref. 36.

## Supplementary Material

Supplementary File

Supplementary File

Supplementary File

## Data Availability

All custom MATLAB scripts reported in this article are available at https://github.com/Neurobiophysics. All study data are included in the article and/or supporting information.
