# Supplementary material for: Real-time visualization of mRNA synthesis during memory formation in live mice
Source: Proc Natl Acad Sci U S A. 2022 Jul 1;119(27):e2117076119. doi: 10.1073/pnas.2117076119 (PMC9271212; doi:10.1073/pnas.2117076119)
Supplement: Supplementary File [file pnas.2117076119.sapp.pdf]

Supplementary Information for

Real-time visualization of mRNA synthesis during memory formation in live mice

Byung Hun Lee, Jae Youn Shim, Hyungseok C. Moon, Dong Wook Kim, Jiwon Kim, Jang Soo Yook, Jinhyun Kim, and Hye Yoon Park*

*Corresponding author.

Email: hyp@umn.edu

This PDF file includes:

Author Contributions

Supplementary Materials and Methods

Figures S1 to S17

Legends for Movies S1 to S2

SI References

Other supplementary materials for this manuscript include the following:

Movies S1 to S2

Author Contributions

J.Y.S. and H.Y.P. developed the PCP-GFP mouse model. B.H.L. and J.Y.S. performed CFC and *in vivo* imaging of *Arc* transcription. B.H.L. and D.W.K. performed VR experiments with *in vivo* imaging of calcium activity and *Arc* transcription. B.H.L. developed the image-processing algorithms. B.H.L., J.Y.S., H.C.M., and D.W.K. performed the image analysis. H.C.M. and J.Y.S. performed the Western blot analysis. J.K., J.S.Y., and J.K. performed smFISH, immunofluorescence, and GFP imaging in fixed brain slices. B.H.L. and H.Y.P. wrote the paper. H.Y.P. conceived and supervised the project.

Supplementary Materials and Methods

Western blotting

Six-week-old female mice were housed in the home cage for 7 days and habituated by daily handling. On the eighth day, contextual fear conditioning (CFC) was performed in Context A. After CFC, the mice were housed in the home cage for 1 hour and sacrificed by cervical dislocation after isoflurane anesthesia. Brain tissue extracts were prepared using T-PER tissue protein extraction reagent (Thermo Fisher Scientific, 78510) containing 1× protease inhibitor (Roche, 04693159001). 30 μg aliquots of protein were separated on 4-12% Bis-Tris polyacrylamide precast gels (Invitrogen, NW04120BOX) in MES-SDS running buffer (Invitrogen, B0002) and transferred to nitrocellulose membranes by using the Mini Blot Module (Thermo Fisher Scientific) following the manufacturer’s instructions. The following antibodies were used: Anti-Arc (1:200, Santa Cruz Biotechnology, sc-17839) and anti-GAPDH (1:100,000, Sigma, G9545) as primary antibodies, and anti-rabbit IgG conjugated to HRP (1:5,000, SA002, GenDEPOT), and anti-mouse IgG conjugated to HRP

(1:5,000, SA001, GenDEPOT) as secondary antibodies. Western blots were scanned by LAS 4000 (GE Healthcare Life Sciences). The images were analyzed by Image Studio Lite Ver 5.2 (LI-COR Biosciences).

Immunofluorescence

12–13-week-old male mice were housed in the home cage and habituated by handling for 3 days. Then, the mice were subject to CFC and returned to the home cage for 1.2–1.4 hr. After isoflurane anesthesia, the animals were perfused transcardially with 15 ml of cold phosphate-buffered saline (PBS) containing 10 U/ml heparin (Sigma, H3393) for 3–4 min and 30 ml of cold 4% PFA in 0.1 M PBS for 7-8 min. Brains were post-fixed in 4% PFA in 0.1 M PBS at 4 °C for more than 4 hr and embedded in 3% agarose gel in 0.1 M PBS. With a vibratome (Leica, VT1000s), brains were sectioned coronally to 50 μ m, permeabilized with 1% Triton X (Thermo Fisher Scientific, 28314) in Tris-buffered saline (TBS) for 30 min at room temperature (RT), and incubated in 0.4% Triton X and 5% normal goat serum (NGS) (Vector Labs, S-1000) in TBS for 30 min at RT. Sections were treated with primary antibodies in 0.4% Triton X and 5% NGS in TBS at 4 °C overnight and then washed three times with 0.1% Triton X in TBS for 20 min at RT. After secondary antibody treatment for 3 hr at RT, the sections were counterstained with 1 μg/ml DAPI (Thermo Fisher Scientific, 62248) in 0.1% Triton X in TBS. Then, the sections were washed with 0.1% Triton X in TBS for 20 min at RT and then twice with TBS for 20 min, and cover-slipped with VectaShield mounting media (Vector Labs, H-1700). Anti-Arc (1:1000, Synaptic Systems, 156 003) was used as primary antibody, and goat-anti rabbit IgG conjugated to Alexa Fluor 555 (A21428; 1:200) was used as secondary antibody.

Imaging was performed using a confocal laser scanning microscope (Carl Zeiss, LSM710) with a 40× 1.2 NA objective (Carl Zeiss, 421767-9971-790).

For immunofluorescence in cultured neurons, we stimulated neurons with 50 µM bicuculline and imaged neurons at 3 min interval for 1.5 hours. Anti-Arc (1:1000, Synaptic Systems, 156 003) was used as primary antibody, and goat-anti rabbit IgG conjugated to Alexa Fluor 647 (A21245, 1:500) was used as secondary antibody. Imaging was performed using a wide-field microscope (Olympus, IX83) equipped with a 150× 1.45 NA oil immersion objective (Olympus, UAPON150XOTIRF) and an EMCCD camera (Andor iXon Life 888).

Simulations

To compare the accuracy of GERI and conventional IEG promoter-based reporter expression methods for identifying IEG-positive neurons, we generated random *Arc* activation traces for 500 neurons. At the time of stimulation, 25% of the neurons were activated during 4 min to mimic the CFC experiment. The basal level of IEG activation was varied from 0–0.5% per min. The IEG activation traces were then convolved with the response function of GERI or that of shGFP reporter (1). The response function of GERI was obtained from the average intensity profile of the *Arc* transcription sites (n = 42) over time. The response function of shGFP was constructed from data in a previous report (2). The fraction of IEG-positive neurons was calculated by counting the number of neurons in which the intensity exceeded a threshold of 30% of the peak intensity at 6 min after stimulation for GERI and at 1 hr after stimulation for shGFP, respectively. The simulation was repeated 10 times. The accuracy was calculated by dividing the number of correctly

predicted neurons by the number of total neurons (n = 500).

Electrical stimulation of cultured hippocampal neurons

Electrical burst stimulation was applied to cultured hippocampal neurons through two thin platinum wires. Hippocampal neurons were cultured from postnatal day 1 (P1) pups of WT mice, which were infected with AAV-hSyn-jRGECO1a at 14 days in vitro (DIV), and used for experiments at 17 DIV. Stimulation patterns were generated by an isolated pulse stimulator (A-M Systems, model 2100) and Arduino UNO microcontroller. Each burst consisted of two biphasic pulses with 2 ms duration, 10 ms interval, and 3.7 V amplitude. Ten bursts were applied at 6, 8, and 10 Hz with an interval of 7 s. A wide-field fluorescence microscope (Olympus, IX83) equipped with a 20× 0.5 NA objective (Olympus, UPLFLN20X), a motorized stage (Marzhauser), and an EMCCD camera (Andor iXon Life 888) was used to acquire time-lapse images of jRGECO1a at 30 frame per second (fps) during the electrical stimulation.

Fixed brain imaging

Fixed brain slices were prepared as previously described, with minor modifications (3). Mice were deeply anesthetized with isoflurane and perfused transcardially with 10-15 ml of PBS containing 10 U/ml heparin (Sigma, H3393), and 30-50 ml of fresh 4% paraformaldehyde (PFA, Sigma-Aldrich, 158127) in 0.1 M phosphate buffer (PB). Brains were post-fixed in 4% PFA at 4 °C overnight, and sectioned coronally to 50 μm with a vibratome (Leica, VT1200S). The sections were counterstained with 0.1 μg/ml DAPI (Invitrogen, D1306) in PBS and cover-slipped with VectaShield mounting media (Vector

Labs, H-1400). Imaging was performed using a slide scanner (Zeiss, Axio Scan.Z1) with a 20× 0.8 NA objective (Zeiss, 420650-9902-000) or a confocal microscope (Zeiss, LSM780) with a 40× 1.3 NA oil immersion objective (Zeiss, 420460-9900-000).

To image jRGECO1a in fixed brain tissues, we used a wide-field fluorescence microscope (Olympus, IX83) equipped with a 20× 0.5 NA objective (Olympus, UPLFLN20X), a motorized stage (Marzhauser), and an EMCCD camera (Andor iXon Life 888). To obtain a large field of view, we performed 15 × 18 grid imaging and stitched the images after shading correction using BaSiC software (4).

Single-molecule fluorescence *in situ* hybridization (smFISH)

smFISH was performed using an RNAscope fluorescent multiplex assay (ACDBio) according to the manufacturer's protocol. After CFC, brains were harvested after decapitation and immediately frozen in -80 °C ethanol. The brains were sectioned coronally to 20 μm using a cryostat (Thermo Fisher Scientific, HM525 or Leica, CM1860) and collected on Superfrost microscope slides (Thermo Fisher Scientific, J1800AMNZ). The sections were fixed in 4% PFA at 4 °C for 15 min. Following serial dehydration in 50%, 70%, and 100% ethanol at RT, the sections were incubated in 100% ethanol overnight at -20 °C. Sections were then treated with proteinase IV for 30 min at RT and rinsed with 0.1 M PBS. Probes were applied for 2 h at 40 °C. Sections were incubated with probes for 2 h at 40 °C and subsequently incubated with amplifiers 1–4 at 40 °C. After counterstaining with DAPI solution, the sections were cover-slipped with ProLong mounting media (Invitrogen, P36965). Images were obtained using a Zeiss slide scanner with a 20× 0.8 NA objective or a confocal microscope (Zeiss, LSM780) with a 40× 1.3 NA oil immersion

objective (Zeiss, 420460-9900-000). Probes used in this study were Arc-C1 (Cat# 316911), PP7-C2 (Cat# 300031, custom-designed), c-Fos-C2 (Cat# 316921), and Egr-1-C3 (Cat# 423371).

Place cell identification

Place cells were defined using a method similar to an approach described previously (*3*). The 3-m-long virtual track was first divided into 150 position bins (2 cm per bin). Using the ΔF/F traces obtained during running (speed >1.5 cm/s and run length > 5 cm), we calculated the sum of ΔF/F for each bin and normalized by the dwell time in each bin to generate a ΔF/F field map. The resulting ΔF/F field map was smoothed by computing the moving average with a sliding window of three bins. The potential place field was identified as a region exceeding the median value of the ΔF/F field map. Place cells were then identified using the following criteria: 1) the potential field is wider than 20 cm; 2) the mean value of the ΔF/F field map in the potential field must be 2.2 times higher than the mean value of the ΔF/F field map outside the potential field; and 3) Ca^{2+} events must be present in at least 15% of the visits to the potential field. To evaluate whether each mouse actually distinguished virtual Contexts A and B, we calculated spatial correlation. For neurons identified as place cells on day 1, we calculated the Pearson's correlation coefficient of the ΔF/F field map between the same or different contexts. The spatial information was calculated using the following formula (5):

$$\text{Spatial information} = \sum_i \frac{\lambda_i}{\lambda} \log_2 \frac{\lambda_i}{\lambda} P_i$$

where λ is the Ca^{2+} event rate, λ_i is the mean Ca^{2+} event rate in the ith place bin, and P_i is

the probability that the mouse stays in the ith place bin.

Network graph

We first converted the Ca^{2+} event traces into binary and temporally binned (1 sec per bin) traces. The resulting traces were used to generate a correlation matrix by calculating Pearson's correlation coefficients. We then generated a binary adjacent matrix with pairs that were statistically significant ($p < 0.05$) and had a correlation coefficient higher than 0.1. The network graph was visualized using Gephi (https://gephi.org/) software with the ForceAtlas2 layout. We calculated degrees, cluster coefficients, and modularity using custom written MATLAB scripts. The degree k_v denotes the number of edges in each neuron. The normalized degree was calculated by dividing k_v by (the number of neurons in network -1). Clustering coefficients were calculated using the following formula (6):

$$C_v = \frac{\sum_{j,k} A_{vj} A_{jk} A_{kv}}{k_v (k_v - 1)}$$

where A is the adjacent matrix in which A_{ij} is a binary value indicating whether the ith neuron and the jth neuron are correlated. The normalized modularity was calculated using the following formula (7):

$$Q_{normalized} = \frac{Q}{Q_{max}} = \frac{\sum_{i,j} \left(A_{ij} - \frac{k_i k_j}{2m} \right) \delta(t_i, t_j)}{2m - \sum_{i,j} \frac{k_i k_j}{2m} \delta(t_i, t_j)}$$

where m is the number of edges in the network, t_i denotes the module (*Arc*++/non-*Arc*++ or place cell/non-place cell) of the ith neuron, and δ is a delta function.

Supplementary Figures

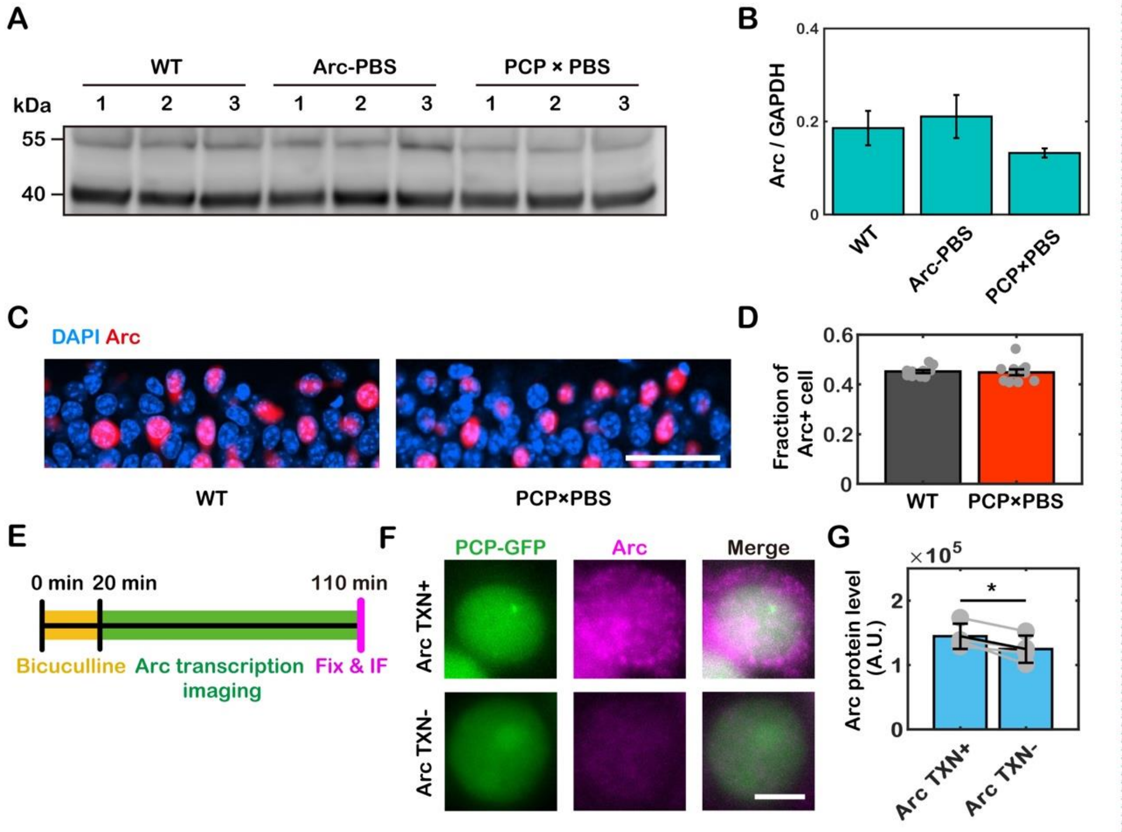

Fig. S1. Comparison of Arc protein expression levels. (A) Western blot of Arc (55 kDa) and GAPDH protein (36 kDa) in brain tissue lysates of three mice from each group of wild type (WT), homozygous Arc-PBS knock-in (Arc-PBS), and double homozygous Arc-PBS knock-in × PCP-GFP (PCP×PBS) at 1.5 hours after CFC. **(B)** Quantification of relative Arc protein expression levels using GAPDH as a loading control. No significant differences were observed between the mouse lines (n = 3 mice for each line). Error bars represent standard deviation (SD). **(C)** Example images of Arc immunofluorescence (red) in dorsal CA1 of WT (left) and PCP×PBS mice (right). The immunofluorescence was performed at 1.5 hours after CFC. Scale bar, 50 µm. **(D)** The fraction of Arc-expressing cells in WT and PCP×PBS mice (n = 12 slices from two mice for each mouse line). **(E)**

Dissociated hippocampal neurons cultured from PCP×PBS mice were stimulated by bicuculline treatment for 20 min. After identifying neurons with *Arc* transcription sites, immunofluorescence (IF) was performed 90 minutes after stimulation. **(F)** Example images of Arc immunofluorescence (magenta) in a neuron with *Arc* transcription (Arc TXN+, upper) and a neuron without *Arc* transcription (Arc TXN-, lower). Scale bar, 5 µm. **(G)** The intensity of Arc protein in Arc TXN+ and Arc TXN- neurons (n = 4 independent experiments, * $P < 0.05$, by pairwise *t* test). Error bars represent the standard error of the mean (SEM).

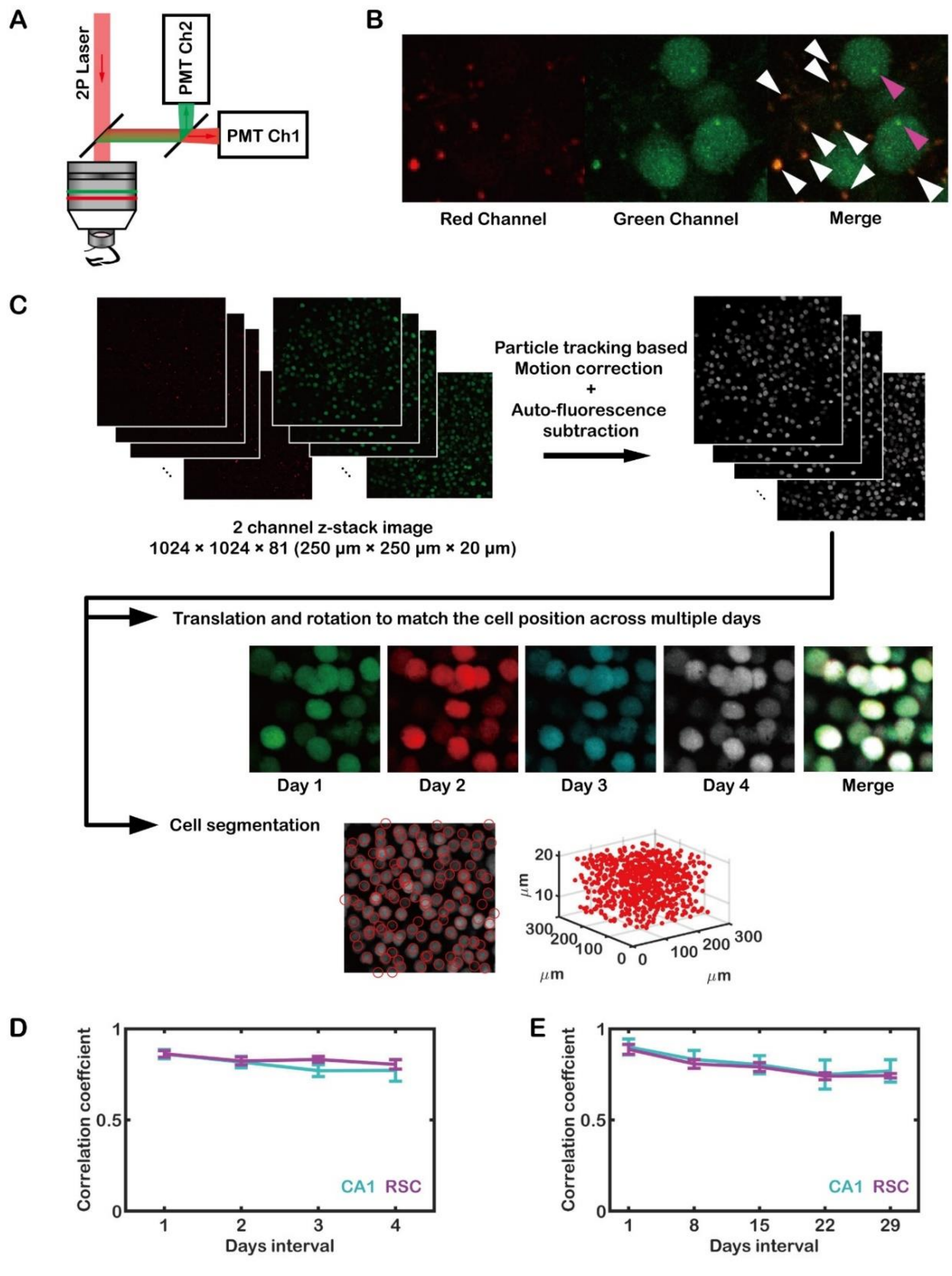

Fig. S2. Image processing procedures. (A) Two-photon microscopy for dual-color *in vivo* imaging. **(B)** Raw images taken by two-photon microscopy. Auto-fluorescent particles (white arrow) were observed in both green and red channels. *Arc* transcription sites

(magenta arrow) were detected only in the green channel. **(C)** Motion artifacts were corrected by a custom particle-tracking algorithm. Auto-fluorescent signals were subtracted and the images were registered across multiple imaging sessions by translation and rotation. Finally, automatic cell segmentation was performed to find neuronal coordinates. **(D-E)** Pearson's correlation coefficients were calculated between images taken 1–4 days apart (D) and 1–4 weeks apart (E). Error bars represent the SEM.

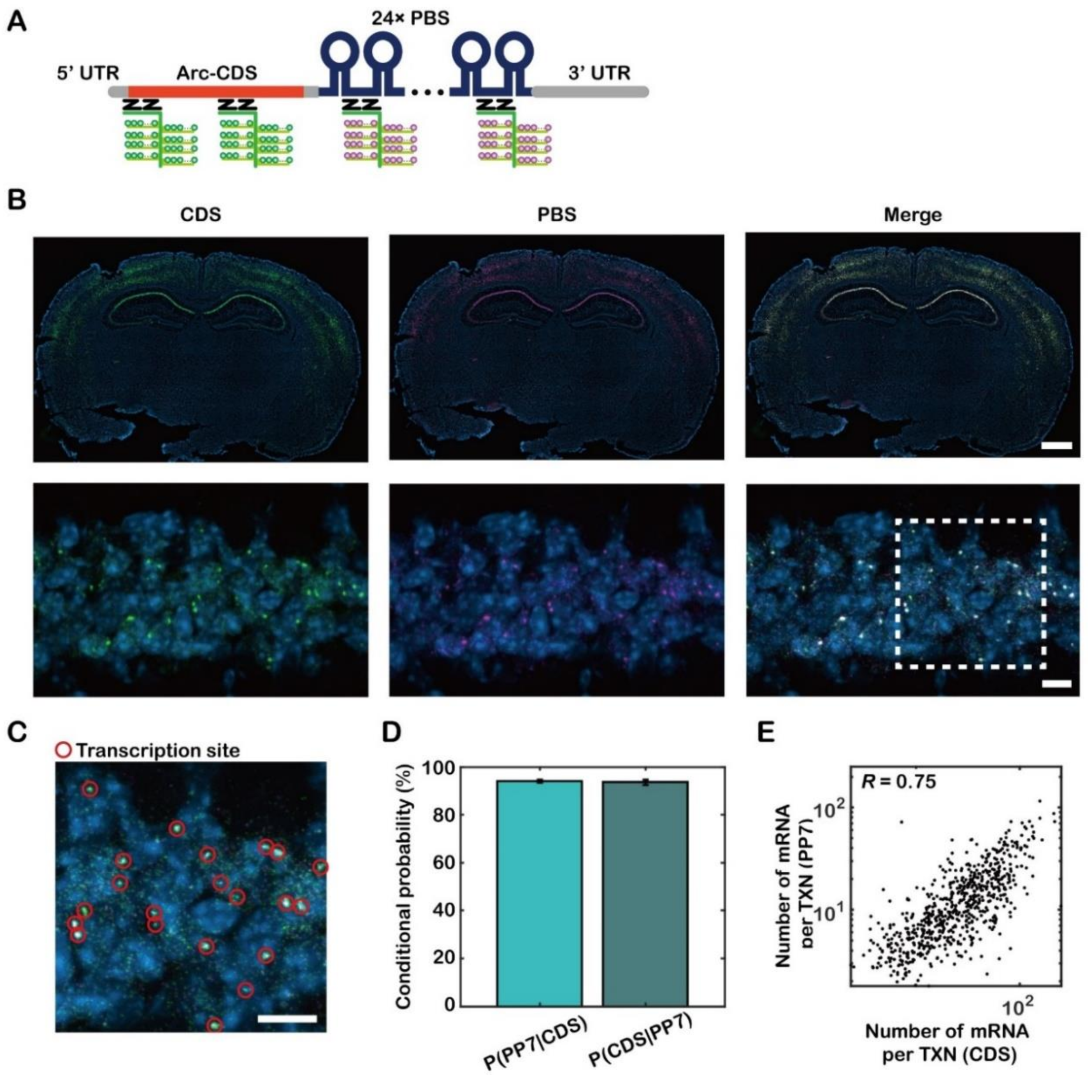

Fig. S3. Dual-color single-molecule fluorescence *in situ* hybridization (smFISH) targeting the CDS and PBS region of *Arc* mRNA. (A) Schematic for dual-color smFISH. The CDS region of *Arc* mRNA was detected by Atto 550 dye (green) and PBS was detected by Atto 647 dye (magenta). **(B)** Representative smFISH images. **(C)** An enlarged image of the dotted box in (B). Red circles denote transcription sites. **(D)** Conditional probability of detecting transcription sites in different channels. **(E)** Scatter plot of the number of nascent mRNAs per transcription site detected by the probes targeting CDS and PBS (n = 670

transcription sites, correlation calculated by Spearman's coefficient *R*). Scale bars, (B, upper panels) 1 mm and (B, lower panels and C) 10 μm.

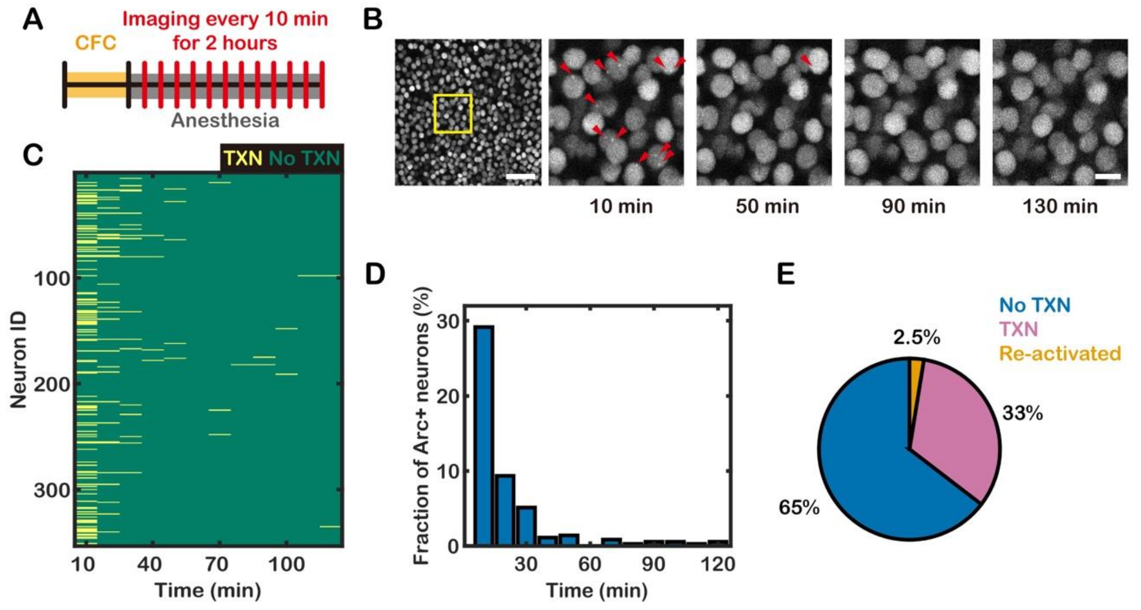

Fig. S4. ***Arc*** **transcriptional dynamics after CFC. (A)** After CFC, time-lapse images of CA1 neurons were acquired every 10 min for 2 hours in an anesthetized mouse. **(B)** Time-lapse images showing the decay of *Arc* transcription sites. Red arrows indicate *Arc* transcription sites. Scale bars, 50 μm (the first image) and 10 μm (the last image). **(C)** *Arc*+ transcriptional on (yellow) and off (green) states are shown over time for individual neurons. **(D)** The fraction of *Arc*+ neurons over time. **(E)** The percentage of neurons that had *Arc* transcription (TXN) only once (pink), neurons that re-activated *Arc* TXN (orange), and neurons that never showed *Arc* TXN (blue) within 2 hours after CFC.

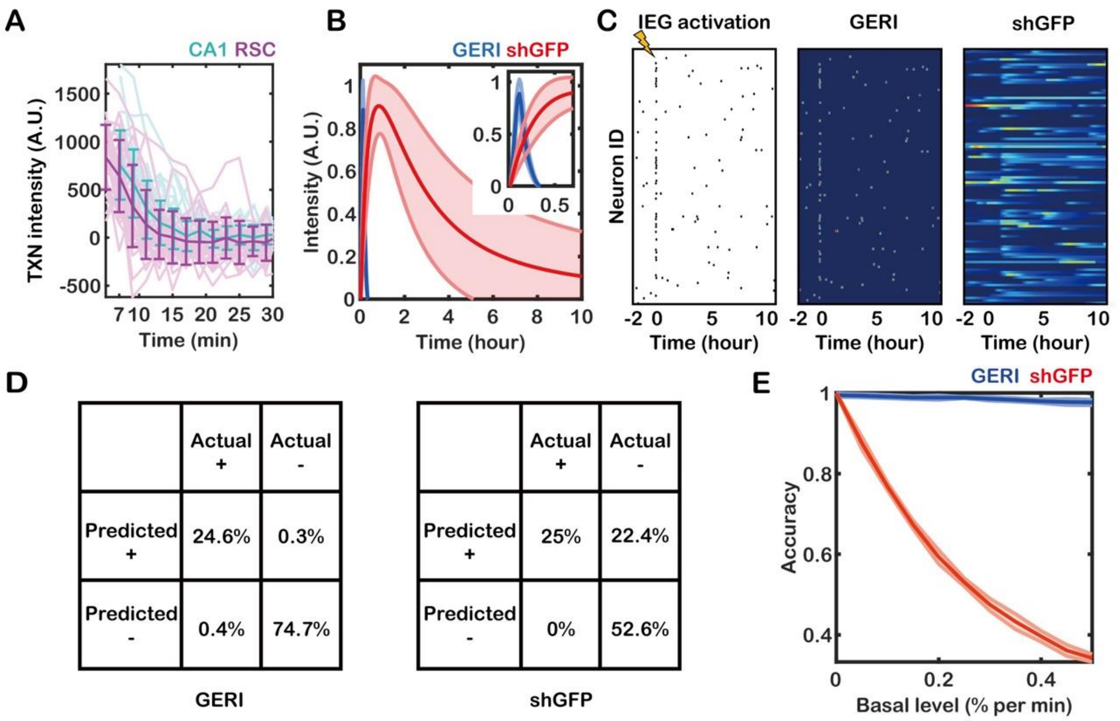

Fig. S5. Simulation of GERI and shGFP reporter-based detection of IEG+ neurons. **(A)** The intensity changes of *Arc* TSs over time in CA1 (cyan, *n* = 48 TSs) and in the RSC (magenta, *n* = 31 TSs). Error bars represent mean ± SD. The transcription sites disappeared with similar half-lives in both CA1 (2.9 ± 0.3 min) and RSC neurons (2.7 ± 0.3 min). **(B)** The response functions of GERI (blue) and shGFP (red) over time. The inset shows a magnified view of the first hour. **(C)** Example images of simulated IEG activation traces (left), and intensity traces generated by convolution of the activation trace and the response function of GERI (middle) and shGFP (right). The probability of IEG activation was 0.1% per min in the baseline and 25% in 4 min (6.25% per min) during stimulation (lightning symbol). **(D)** Confusion matrices showing the classification results using GERI (left) and shGFP (right) when the basal IEG activation probability was 0.1% per min and stimulated

activation probability was 25% in 4 min. **(E)** The accuracy of GERI (blue) and shGFP (red) methods plotted for different basal levels of IEG activation.

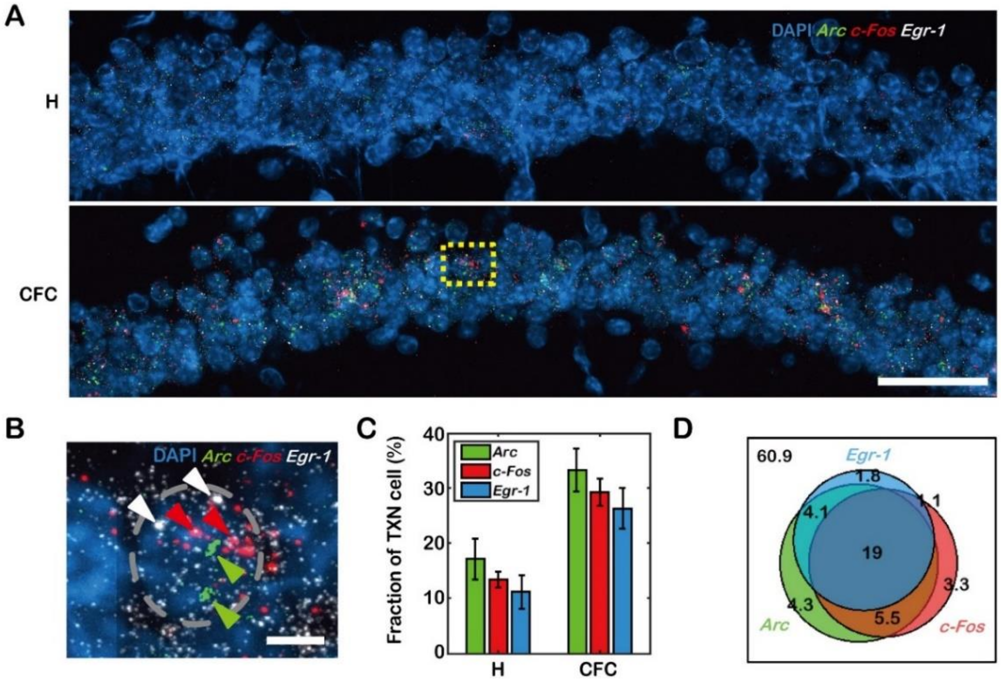

Fig. S6. Three-color smFISH targeting *Arc*, *c-Fos* and *Egr-1* mRNA in CA1. (A) Representative smFISH images of hippocampi from mice held in the home cage (H; upper panel) and after CFC (lower panel). *Arc, c-Fos* and *Egr-1* mRNA were labeled with FITC (green), Atto 550 (red), and Atto 647 (white) dyes, respectively. **(B)** Enlarged image of yellow box in (A). White, red, and green arrows indicate transcription sites of *Egr-1*, *c-Fos*, and *Arc*, respectively. **(C)** The fraction of cells with a transcription site for each gene. **(D)** A Venn diagram showing the percentage of cells expressing each gene. Scale bars, (A) 50 µm and (B) 5 µm. Error bars represent the SEM.

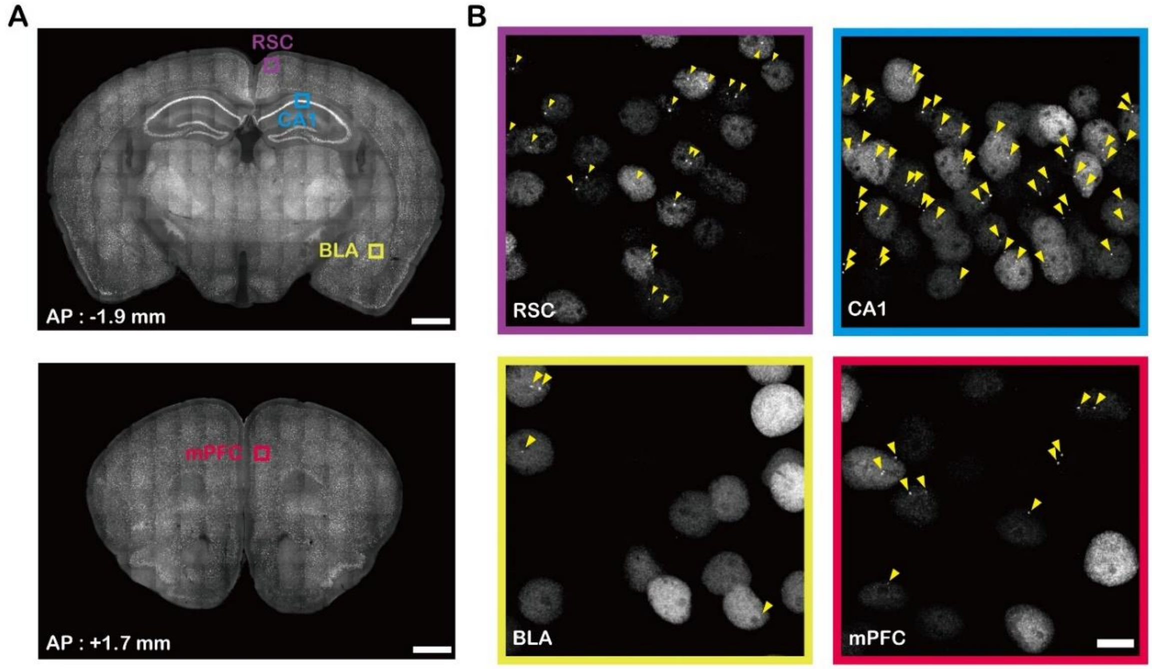

Fig. S7. GFP images of fixed brain slices. (A) Coronal sections of a PCP×PBS mouse brain at two AP positions (AP = -1.9 mm and +1.7 mm). Upper panel includes retrosplenial cortex (RSC), CA1, and basolateral amygdala (BLA). Lower panel includes medial prefrontal cortex (mPFC). **(B)** Enlarged images of the inset boxes in (A). Scale bars, (A) 1 mm and (B) 10 μm.

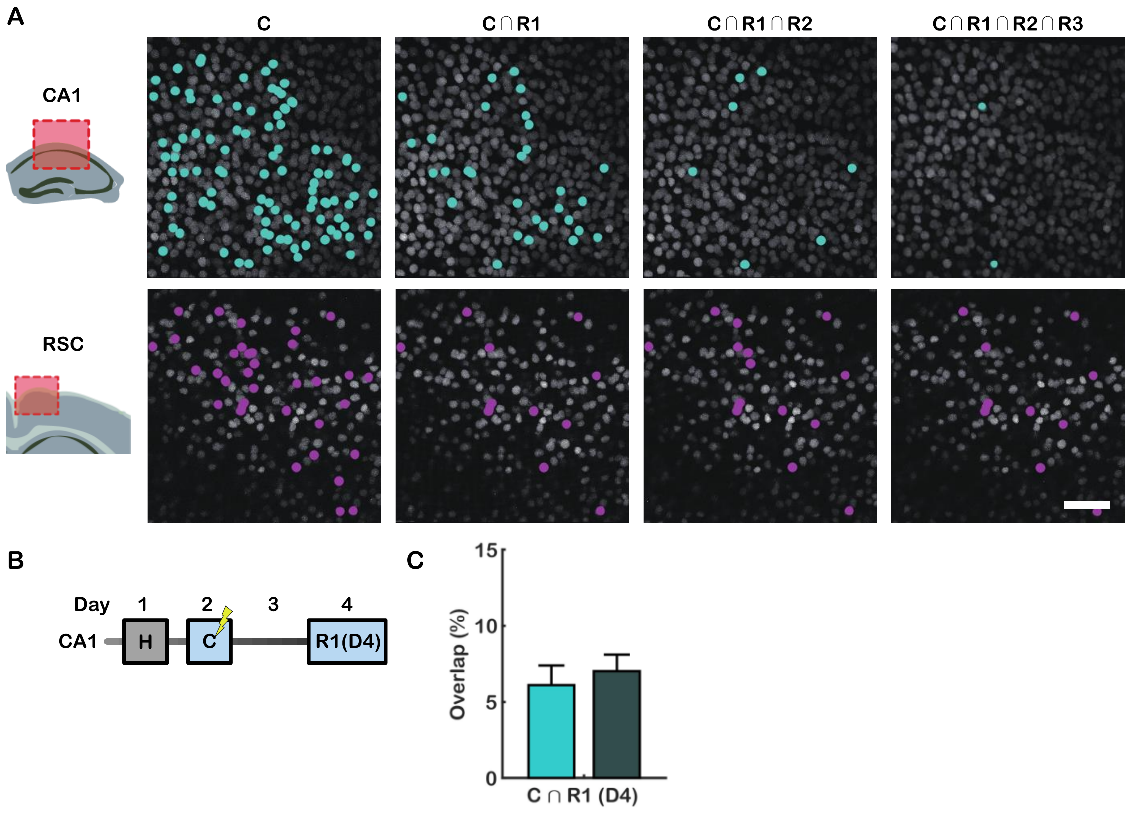

Fig. S8. Overlap of *Arc*+ neurons upon recent memory retrieval. **(A)** Representative images of CA1 (upper panels) and the RSC (lower panels) showing overlapping populations of *Arc*+ neurons upon recent memory retrievals. (cyan dots, CA1; magenta dots, RSC). Scale bar, 50 μm. **(B)** Experimental scheme for the first fear memory retrieval test at two days after CFC (R1(D4)). **(C)** Overlap percentage of *Arc*+ neurons between the CFC and R1(D4) conditions (n = 4 mice). Error bars represent the standard error of the mean (SEM).

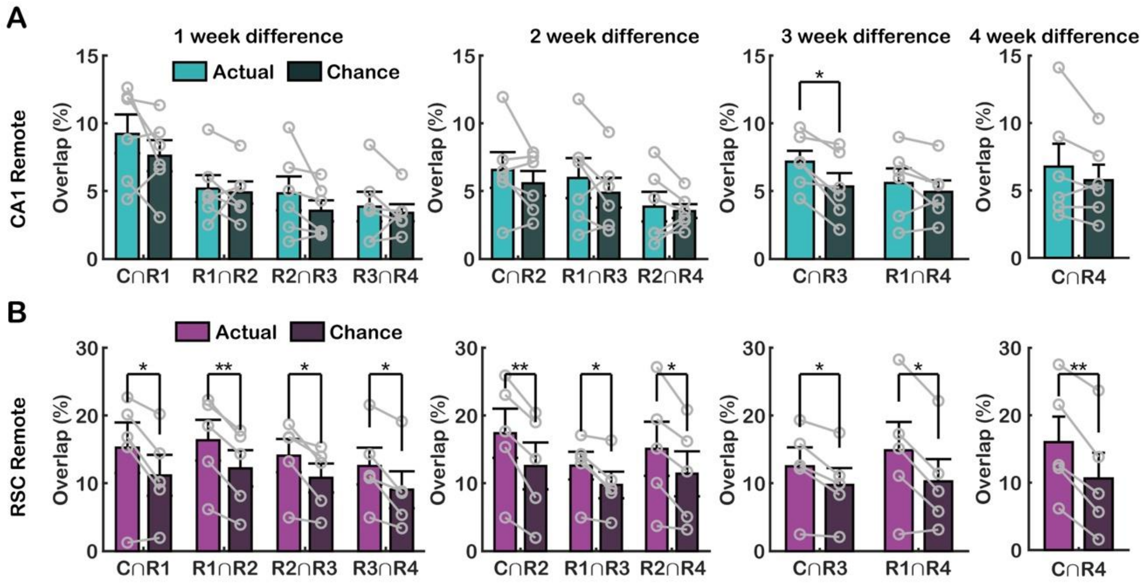

Fig. S9. Overlap between *Arc*+ populations after remote memory retrievals (A-B) The percent overlap between *Arc*+ ensembles in CA1 (A; n = 6 mice) and the RSC (B; n = 5 mice), with 1–4-week differences compared with the chance level (* $P < 0.05$, ** $P < 0.01$ by pairwise t test). RSC ensemble overlap was generally significantly greater than chance levels, whereas most CA1 ensembles exhibited chance-level overlap. Error bars represent the SEM.

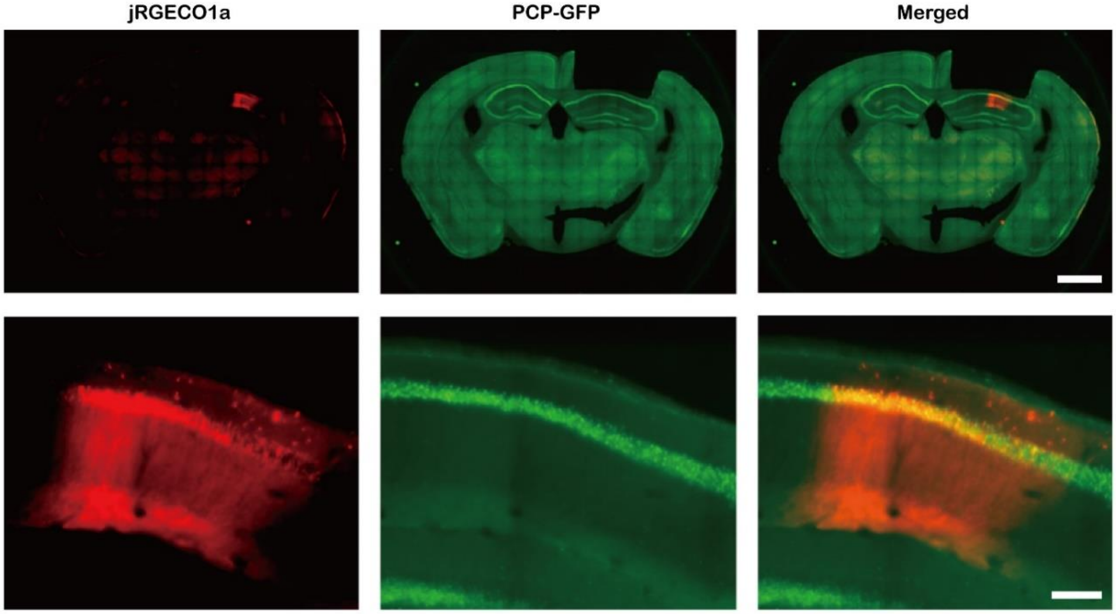

Fig. S10. Expression of jRGECO1a in CA1 of PCP×PBS mouse. AAV1-hSyn-NES-jRGECO1a was injected into the dorsal CA1 of PCP×PBS mice**.** Coronal section images of the dorsal CA1 pyramidal cell layer expressing jRGECO1a (red) and PCP-GFP (green). Scale bars, (upper panel) 1 mm and (lower panel) 100 μm.

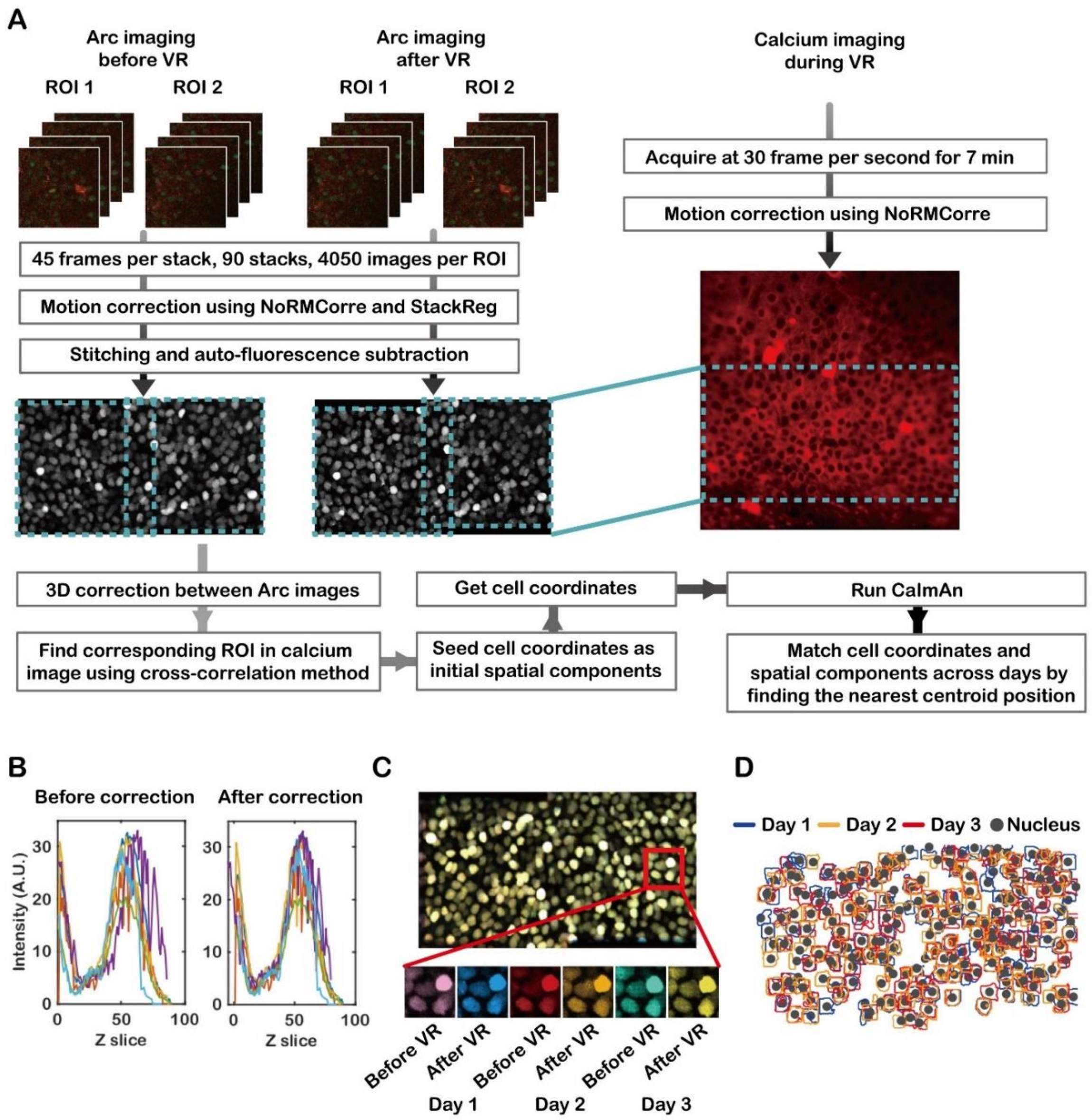

Fig. S11. Image registration pipeline for *Arc* mRNA and calcium images. (A) Raw images of *Arc* mRNA were first corrected for motion artifacts, followed by ROI stitching, auto-fluorescence subtraction, correction across days, and cell coordinate detection. Calcium images were corrected for motion artifacts, and calcium footprints were detected using constrained non-negative matrix factorization (CNMF) with information of detected cells (see Methods for details). **(B)** A z-slice profile of *Arc* mRNA images before (left) and after correction (right). **(C)** Corrected images of *Arc* mRNA across multiple days. **(D)**

Identified spatial footprints using CNMF over multiple days. Cell coordinates and spatial footprints were matched by finding the nearest centroid position.

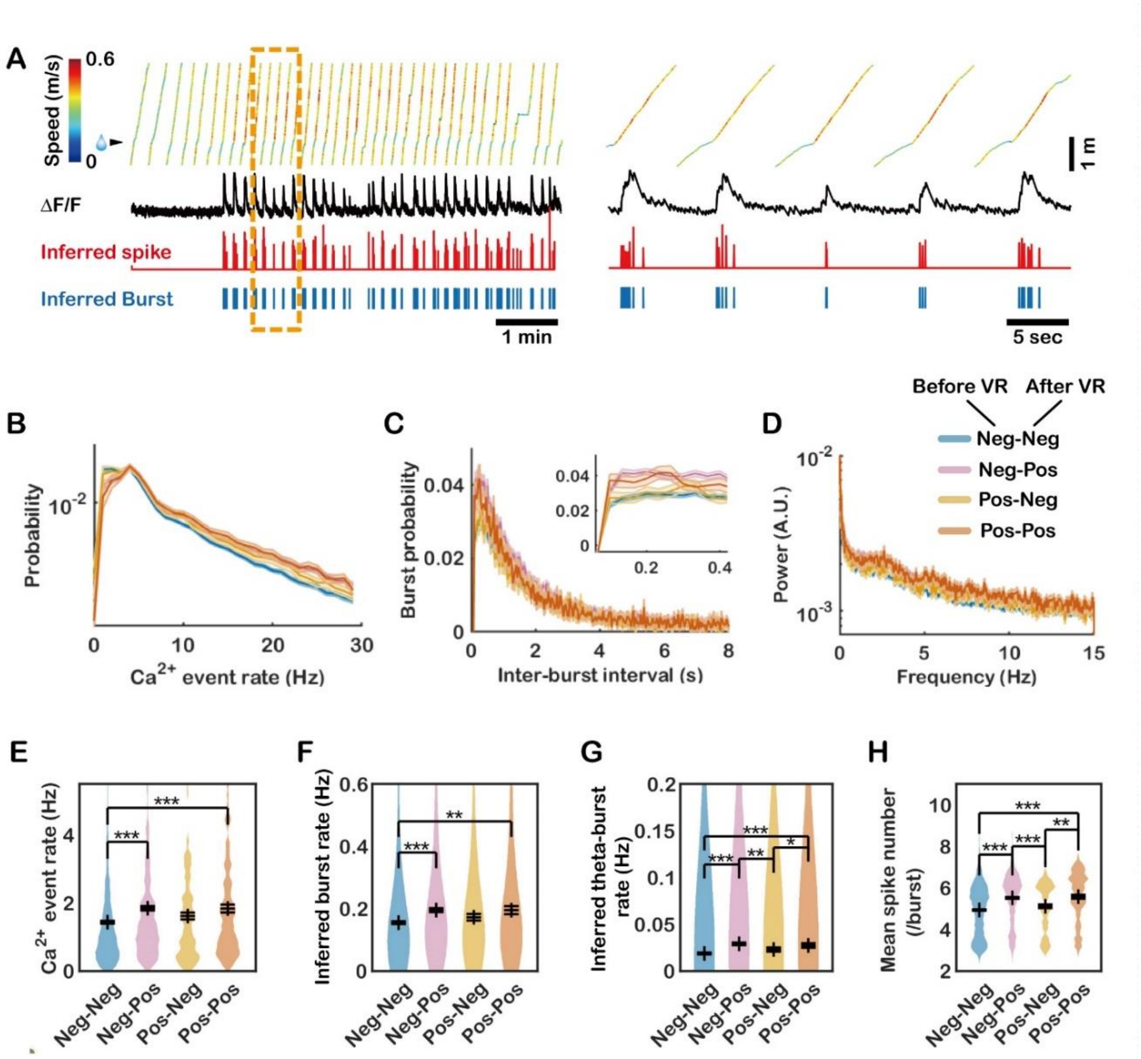

Fig. S12. Calcium activity of CA1 neurons during VR navigation. (A) An example of the VR track position with the colormap of the running speed over time. A representative neuron's calcium trace, inferred spike trace from deconvolution, and inferred burst trace are shown at the bottom (left). A segment of traces in the yellow dashed box is magnified at right. **(B-D)** We classified neurons into four classes according to neuronal transcription of *Arc* mRNA before and after VR. The Ca^{2+} event rate histogram (B), autocorrelation (C), and power spectrum (D) of each class were plotted. **(E-H)** The Ca^{2+} event rate, inferred burst rate, inferred theta-burst rate, and mean spike number per burst of each group were plotted (* $P < 0.05$, ** $P < 0.01$, *** $P < 10^{-10}$ by rank-sum test). Generally, neurons in the

Neg-Pos group had similar activity properties to neurons in the Pos-Pos group. Error bars represent the SEM.

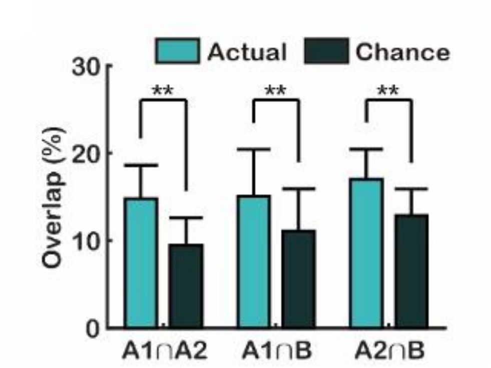

Fig. S13. Comparison of overlap rates with chance levels. Overlap between *Arc*+ neurons identified on days 1, 2, and 3 compared with chance levels (** $P < 0.01$, by pairwise *t* test). Error bars represent the SEM.

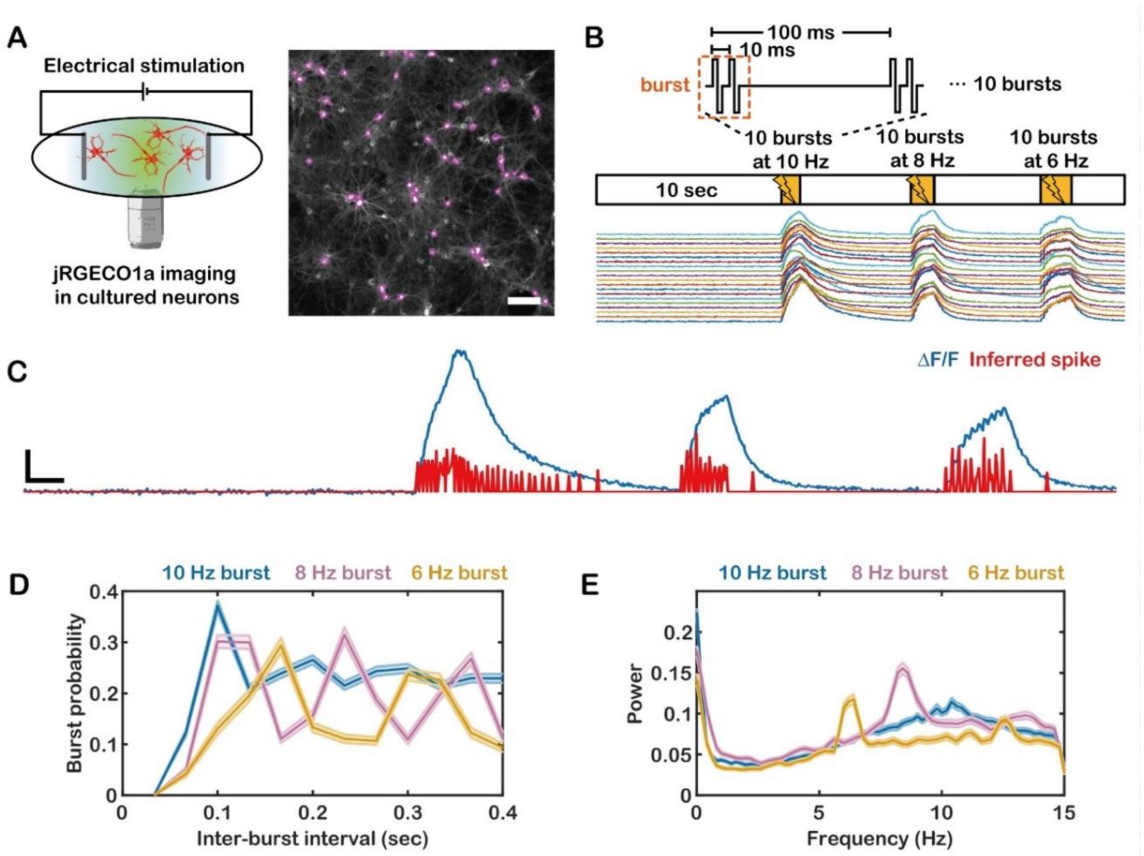

Fig. S14. Calcium imaging of primary cultured hippocampal neurons upon 6–10 Hz burst electrical stimulations. (A) Experimental scheme of electrical stimulation (left) and a representative image of cultured neurons expressing jRGECO1a (right). Magenta contours represent the ROIs for calcium fluorescence signals. **(B)** A single burst contains two electrical pulses with 10 ms interval. 10 bursts were delivered to neurons at 10, 8, and 6 Hz interspaced by 7 s. The calcium traces ΔF/F of 20 representative neurons are shown at the bottom. **(C)** An example ΔF/F trace (blue) and inferred spike trains (red). **(D)** Histogram of inter-burst intervals obtained from the inferred spike trains during 10, 8, and 6 Hz stimulation. **(E)** Power spectra of inferred bursts during 10, 8, and 6 Hz stimulation. Scale bars, (A) 100 µm, (C, vertical) 500% ΔF/F and (C, horizontal) 1 s.

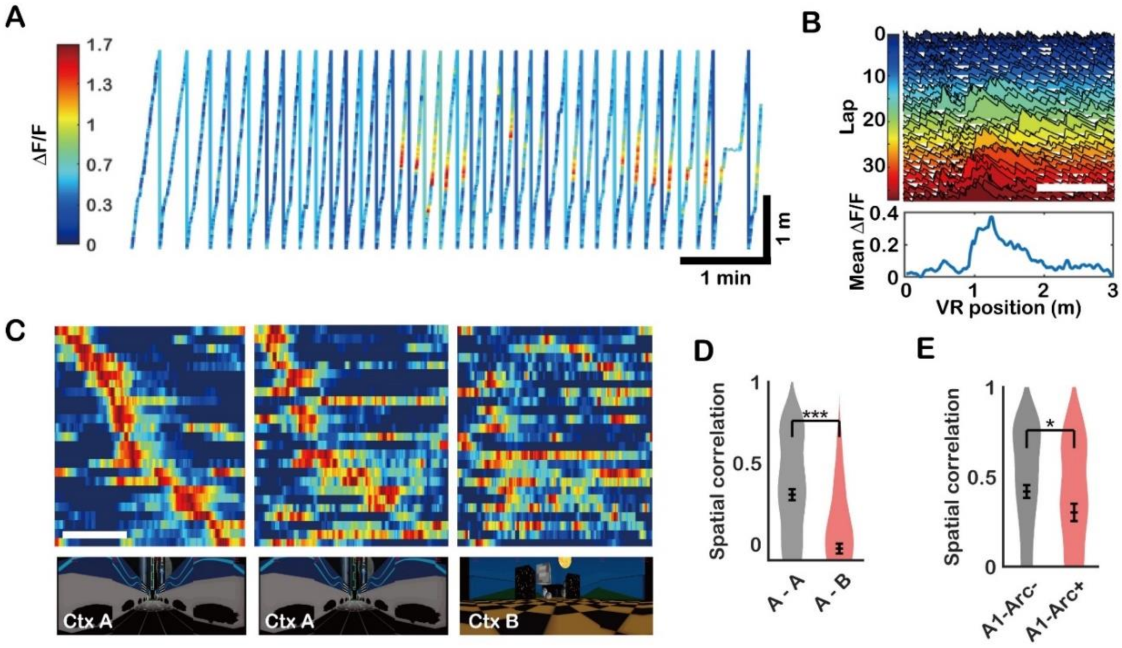

Fig. S15. Spatial correlation after exposure to the same or different virtual contexts. **(A)** Position versus time plot of a mouse running along the virtual linear track, colored according to ΔF/F values of a representative place cell. **(B)** Joyplot showing the calcium traces of each lap and the mean calcium trace of the place cell shown in (A). **(C)** Mean place fields on days 1–3, sorted by place cells detected on day 1. **(D)** Spatial correlation between place fields when mice were exposed to the same (A-A) or different (A-B) contexts. **(E)** Comparison of A1-*Arc*- and A1-*Arc*+ neurons in terms of their spatial correlation between place fields when mice were exposed to the same (A-A) contexts. Scale bar, (C) 1 m.

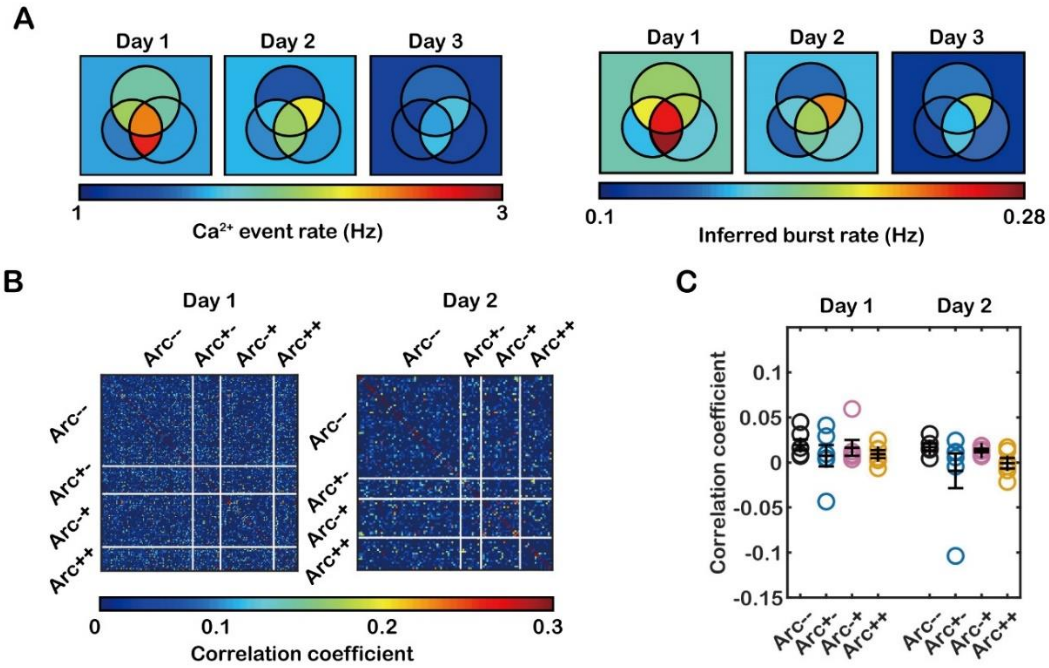

Fig. S16. Ca^{2+} event rates, inferred burst rates and correlation coefficients of *Arc*+ subpopulations. **(A)** Venn diagrams of *Arc*+ neurons on days 1, 2, and 3 colored by the Ca^{2+} event rate (left) and the inferred burst rate (right). **(B)** Correlation matrices generated by calculating the Pearson's correlation coefficient between binned spike traces. The neurons were arranged in the order of Arc--, Arc+-, Arc-+ and Arc++ neurons. **(C)** The correlation coefficient of the binned spike traces of the neurons in each group (n = 6 mice).

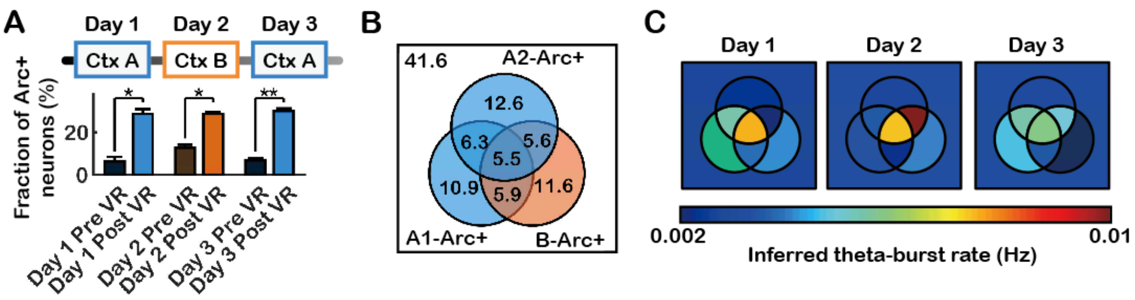

Fig. S17. *Arc* transcription and calcium activity in CA1 upon a different order of exposure to ctx A and B. (A) The fraction of *Arc*+ neurons after VR exploration in ctx A, B, and A on days 1, 2, and 3, respectively (* $P < 0.05$, ** $P < 0.01$ by pairwise *t* test $n = 3$ mice). **(B)** Venn diagram of *Arc*+ neurons on each day. The numbers indicate the fraction of neurons in percentage. **(C)** Venn diagrams are colored by inferred theta-burst rate on each day.

Legends for Supplementary Movies S1 to S2

Movie S1. Three-dimensional view of CA1 in a live PCP×PBS mouse. A scanning volume of 250 µm × 250 µm × 20 µm was imaged in 1024 × 1024 × 81 voxels with a scan speed of 2 µs per pixel.

Movie S2. Time-lapse calcium images of CA1 neurons during VR navigation. The calcium activity of neurons in an area of 256 µm × 256 µm was recorded at 30 fps. The orange contours indicate the *Arc*++ neurons. The movie is played at 16 times real speed.

Supplementary References

1. L. G. Reijmers, B. L. Perkins, N. Matsuo, M. Mayford, Localization of a stable neural correlate of associative memory. *Science* **317**, 1230-1233 (2007).
2. P. Meenakshi, S. Kumar, J. Balaji, In vivo imaging of immediate early gene expression dynamics segregates neuronal ensemble of memories of dual events. *Mol Brain* **14**, 102 (2021).
3. L. Feng, O. Kwon, B. Lee, W. C. Oh, J. Kim, Using mammalian GFP reconstitution across synaptic partners (mGRASP) to map synaptic connectivity in the mouse brain. *Nat Protoc* **9**, 2425-2437 (2014).
4. T. Y. Peng *et al.*, A BaSiC tool for background and shading correction of optical microscopy images. *Nature Communications* **8**, 14836 (2017).
5. B. L. M. W. E. Skaggs, K. M. Gothard, E. J. Markus, An information-theoretic approach to deciphering the hippocampal code. *in Advances in Neural Processing Systems* **5**, 1030-1037 (1993).
6. D. J. Watts, S. H. Strogatz, Collective dynamics of 'small-world' networks. *Nature* **393**, 440-442 (1998).
7. M. E. J. Newman, Modularity and community structure in networks. *Proc. Natl. Acad. Sci. U.S.A.* **103**, 8577-8582 (2006).
